# Bioconjugation of Green Fluorescent Protein via an Unexpectedly Stable Cyclic Sulfonium Intermediate

**DOI:** 10.1002/cbic.201200231

**Published:** 2012-05-25

**Authors:** Ramiz Nathani, Paul Moody, Mark E B Smith, Richard J Fitzmaurice, Stephen Caddick

**Affiliations:** a Department of Chemistry, University College LondonLondon, WC1H 0AJ (UK)

**Keywords:** alkylation, bioconjugation, cysteine, protein modifications, sulfonium

The development of methods for the modification of peptides and proteins under benign aqueous conditions has received a great deal of attention from both industrial and academic laboratories.^1^ The ability to introduce a range of functional post-translational modifications, either natural or synthetic, to recombinantly expressed proteins, such as sugars, lipids, fluorophores, affinity tags or radionuclides, has been exploited to gain further insight into a plethora of biological process.^2^ A number of strategies for protein modification through manipulation of the amino or carboxy terminus have been developed.^3^ However, the vast majority of approaches rely on the reactivity of naturally occurring amino acid side chains such as lysine, tyrosine or cysteine.^4^

**Scheme 1:**
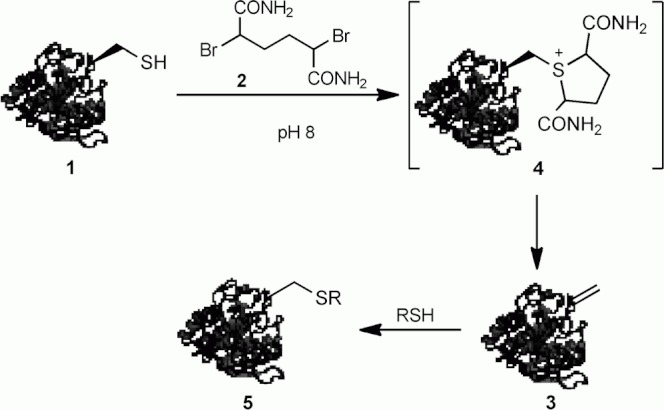
Modification of SBL(S156C) by cysteine β-elimination.

One bioconjugation approach exploits the elimination of cysteine to dehydroalanine, which can then be modified by adding suitable nucleophiles. A number of reagents have been developed to convert both peptide- and protein-based cysteines to dehydroalanine, for example *O*-mesitylenesulfonylhydroxylamine and hexamethylphosphorous triamide.4b, 5 However, the majority of currently used reagents require harsh conditions that limit their application. Recently, Davis has described the elegant use of 1,4-bishaloalkanes for the β-elimination of cysteine to effect protein modification under mild conditions.^6^ An illustrative example is the treatment of a single cysteine mutant of subtilisin from *Bacillus lentus* SBL(S156C) (**1**) with 2,5-dibromohexanediamide (**2**) at pH 8, which afforded intermediate dehydroalanine **3**, presumably via the transient formation of sulfonium **4** (Scheme 1). Dehydroalanine **3** could be subsequently functionalised with a nucleophile to give modified protein **5**.

Intrigued by the potential application of the Davis double-alkylation methodology, we explored its applicability to a range of biological systems. Herein, we report preliminary studies on the modification of a cysteine mutant (S147C) of "superfolder" green fluorescent protein (GFP) bearing a single surface-accessible cysteine. We were able to synthesise unusually stable cyclic sulfonium species and to employ these in a novel bioconjugation that is complementary to current dehydroalanine-based technologies for substrates for which dehydroalanine formation is not facile. Although the presence of sulfonium ions in peptides and proteins derived from the alkylation of methionine is well established,^7^ to our knowledge this represents the first evidence for the formation of a stable cysteine-sulfonium on a protein.

**Scheme 2:**
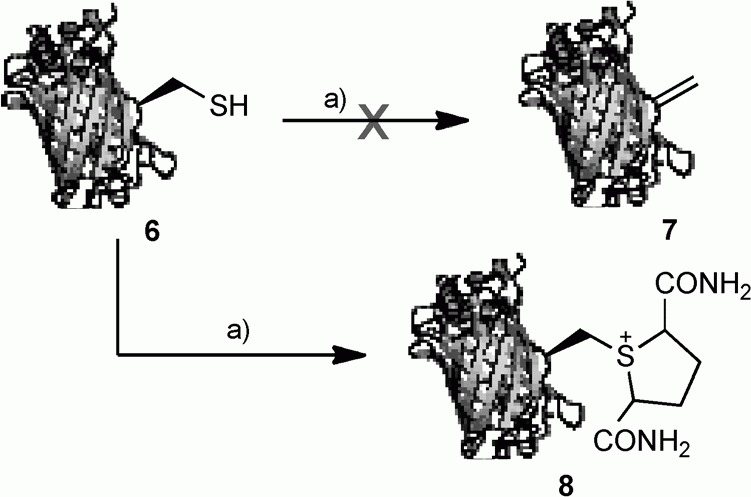
Reaction of GFP(S-147C) (100 μL, 1 mg mL^−1^ in H_2_O, pH 8, 100 mm phosphate) with dibromodiamide (**2**). a) **2** (1500 equiv; 10 μL, 340 mm in DMF), 37 °C, 2 h.

We treated GFP(S147C) (**6**) with **2** under Davis' conditions (1500 equiv, 37 °C, 2 h, pH 8; Scheme 2).^6^ Unexpectedly the product was not the corresponding GFP dehydroalanine **7**, rather electrospray (ES) MS indicated quantitative conversion to a species with a molecular weight equivalent to that of sulfonium **8** (29 486 calcd, 29 488 obs.). Indeed, throughout this study, we observed no evidence for the formation of dehydroalanine **7**, or products derived therefrom, even upon incubation of sulfonium **8** for prolonged periods (vide infra). The isolation of this sulfonium species as a stable adduct and its reluctance to undergo elimination to give the corresponding dehydroalanine product **7** was noteworthy and we embarked upon further studies to understand and exploit this unexpected mode of reactivity.

In order to gain further evidence for the site of reaction between GFP(S147C) and 2,5-dibromohexanediamide, sulfonium **8** was treated with *N*-methylbromomaleimide (1 equiv).4d No reaction was observed with **8**, whereas **6** reacted cleanly, thus suggesting that **2** had reacted exclusively on cysteine to generate **8** (see the Supporting Information).

Optimisation of the formation of **8** was achieved by varying a number of parameters including the number of equivalents of **2**, reaction temperature and reaction time (Table 1). Treatment of GFP(S147C) with 10 equivalents of **2** afforded no sulfonium **8** after 5 h; nevertheless, clear evidence for the formation of thioether **9** (29 566 calcd, 29 563 obs.) was apparent by LCMS (Table 1, entry 2). However, a prolonged incubation time, 20 h, afforded **8** as the only identifiable product in 53 % conversion (Table 1, entry 3). Increasing the concentration of 2,5-dibromohexanediamide afforded, as expected, more rapid formation of **8**, with complete conversion of **6** at 21 °C with 25 or 50 equivalents of **2** within 20 h (Table 1, entries 4–9). At 4 °C, no sulfonium formation is apparent after 20 h even using 25 equivalents of 2,5-dibromohexanediamide (Table 1, entries 10 and 11). However, formation of sulfonium **8** at 4 °C is observed at higher concentrations of **2** (Table 1, entry 12). At 37 °C with 50 equivalents of **2** complete conversion of **6** to **8** could be rapidly achieved (Table 1, entry 13).

**Table 1:** Optimisation of formation of sulfonium 8.^[a]^

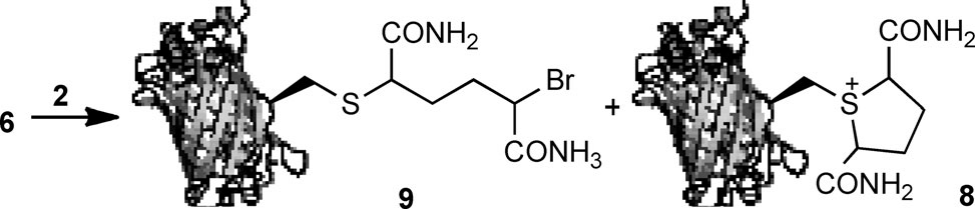
	**2** [equiv]	*T* [°C]	*t* [h]	**6** [%]^[b]^	**9** [%]^[b]^	**8** [%]^[b]^
1	10	21	2	>95	0	0
2			5	91	9	0
3			20	47	0	53
4	25	21	2	81	19	0
5			5	50	17	33
6			20	0	0	>95
7	50	21	2	56	23	21
8			5	27	25	48
9			20	0	0	>95
10	10	4	20	>95	0	0
11	25		20	80	20	0
12	50		20	56	24	20
13	50	37	2	0	0	>95

[a] Conditions: **6** (100 μL, 1 mg mL^−1^ in H_2_O) at pH 8 (100 mM phosphate), **2** (10 μL in DMF). [b] Determined by ratio of peak heights in deconvoluted mass spectrum.

**Scheme 3:**
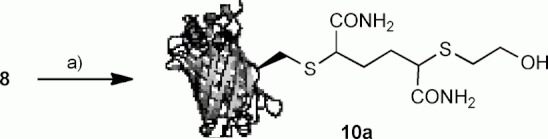
Reactivity of GFP(S147C) sulfonium **8** (100 μL, 0.9 mg mL^−1^ in H_2_O/DMF 10:1, pH 8, 100 mM phosphate) with β-mercaptoethanol (5 μL, 680 mM in H_2_O). a) HSCH_2_CH_2_OH (1000 equiv), 37 °C, 2.5 h, DMF, H_2_O, pH 8 (phosphate), >95 % conversion.

We then sought to establish the stability and reactivity profile of sulfonium **8**. After preparation of sulfonium **8** under our optimised conditions (50 equiv **2**, 37 °C, 2 h) excess **2** could be readily removed by repeated diafiltration into fresh buffer. Sulfonium **8** was found to be stable in the absence of nucleophiles at 21 °C for 24 h and at 4 °C for 1 month; although, significant decomposition was observed at 37 °C after 4 h (vide supra). Treatment of **8** with β-mercaptoethanol (1000 equiv, 37 °C, 2.5 h) afforded bisthioether **10 a** (29 564 calcd, 29 566 obs.) in complete conversion, presumably through ring opening of the cyclic sulfonium (Scheme 3).

We then evaluated the reactivity of **8** with a range of simple nucleophiles. Treatment of **8** with the sodium salt of 1-thio-β-D-glucose (10 equiv) afforded the expected GFP–thioglucose conjugate **10 b** as the sole product, with complete conversion after 5 h (Table 2, entry 1). Similarly, treatment of **8** with glutathione was also successful at both 21 and at 37 °C, generating the corresponding GFP–glutathione adduct **10 c** in excellent conversion (entries 3–5). We also confirmed that these reactions could be carried out effectively at room temperature (21 °C) in 5 h, or more rapidly at 37 °C or with increased concentrations of the nucleophile (entries 2, 4 and 5). Treatment of sulfonium **8** with phenyl selenol (100 equiv) afforded the mixed thio/seleno bisether **10 d** in excellent conversion (entry 6). Although the conversion from reaction between **8** and phthalimide to give **10 e** was only modest (entry 7), we were delighted to observe clean reaction in high conversion upon treatment of **8** with sodium azide (1000 equiv) to generate azide-labelled GFP **10 f** (entry 8).

**Table 2:** Conjugation of nucleophiles with GFP sulfonium 8.^[a]^

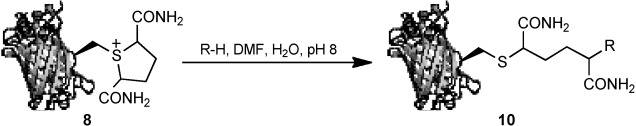
	R–H	Equiv	*T* [°C]	*t* [h]	Conv. **8** [%]^[b]^	Product
1 2	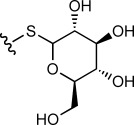	10 100	37 21	5 5	>95 >95	**10 b**
3 4 5	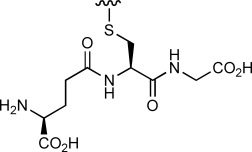	10 100 200	37 21 37	5 5 2	>95 >95 >95	**10 c**
6	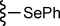	100	37	5	>95	**10 d**
7	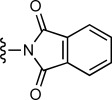	1000	37	2.5	40	**10 e**
8	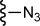	1000	37	2.5	>95	**10 f**

[a] Conditions: **8** (100 μL, 1 mg mL^−1^ in H_2_O) at pH 8 (100 mM phosphate), NuH (see the Supporting Information). [b] Determined by ratio of peak heights in deconvoluted mass spectrum.

In conclusion, we have shown that treatment of superfolder GFP with bis-alkylating agents can be used to prepare highly stable, yet synthetically useful cyclic protein sulfonium adducts. These species undergo clean addition reactions to allow the introduction of a variety of useful chemical motifs. We envisage that the stability of the generated cysteine-derived sulfonium is presumably strongly correlated to the chemical environment of the alkylated cysteine. This new bioconjugation technique could provide a useful entry into multiply functionalised proteins and further work will be directed toward extending these findings to other systems.
