# Supplementary material for: Bioconjugation of Green Fluorescent Protein via an Unexpectedly Stable Cyclic Sulfonium Intermediate
Source: Chembiochem. 2012 May 25;13(9):1283–5. doi: 10.1002/cbic.201200231 (PMC3487180; doi:10.1002/cbic.201200231)
Supplement: Supplementary file 1 [file cbic0013-1283-SD1.pdf]

CHEMBIOCHEM

Supporting Information

© Copyright Wiley-VCH Verlag GmbH & Co. KGaA, 69451 Weinheim, 2012

Bioconjugation of Green Fluorescent Protein via an Unexpectedly Stable Cyclic Sulfonium Intermediate

Ramiz Nathani, Paul Moody, Mark E. B. Smith, Richard J. Fitzmaurice, and Stephen Caddick*[a]

cbic_201200231_sm_miscellaneous_information.pdf

General Procedure

All reagents were purchased from Sigma-Aldrich and used without any purification. Solvents were purchased from Fisher and used without purification. 2,5-Dibromohexanediamide (**2**) was synthesized as reported by Davis.[1]

Protein Mass Spectroscopy

LC-MS was performed on protein samples using a Thermo Scientific uPLC connected to MSQ Plus Single Quad Detector (SQD). Column: Hypersil Gold C4 1.9µm 2.1 x 50 mm. Wavelength: 254 nm. Mobile Phase: 99:1 Water (0.1% formic acid): MeCN (0.1% formic acid) to 1:9 Water (0.1% formic acid): MeCN (0.1% formic acid) gradient over 4 min. Flow Rate: 0.3 mL/min. MS Mode: ES+. Scan Range: m/z = 500-2000. Scan time: 1.5 sec. Data obtained in continuum mode. The electrospray source of the MS was operated with a capillary voltage of 3.5 kV and a cone voltage of 50 V. Nitrogen was used as the nebulizer and desolvation gas at a total flow of 600 L/h. Total mass spectra for protein samples were reconstructed from the ion series using the pre-installed ProMass software using default settings for large proteins in m/z range 500-1500.

Cloning and expression of GFP(S147C) 6

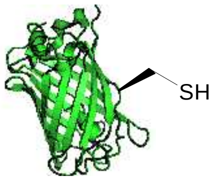

6

Calculated mass = 29343

GFP(S147C) **6** was cloned and expressed as reported by Caddick and analysed by LCMS.[2]

Sequence:

MHHHHHHSSGVDLGTDNLYFQSMRKGEELFTGVVPILVELDGDVNGHKFSVRGEGEGDATNGKLTLKFI
CTTGKLPVPWPTLVTTLTYGVQCFARYPDHMKQHDFFKSAMPEGYVQERTISFKDDGTYKTRAEVKFEG
DTLVNRIELKGIDFKEDGNILGHKLEYNFNCHNVYITADKQKNGIKANFKIRHNVEDGSVQLADHYQQNT
PIGDGPVLLPDNHYLSTQSVLSKDPNEKRDHMVLLEFVTAAGITHGMDELYK

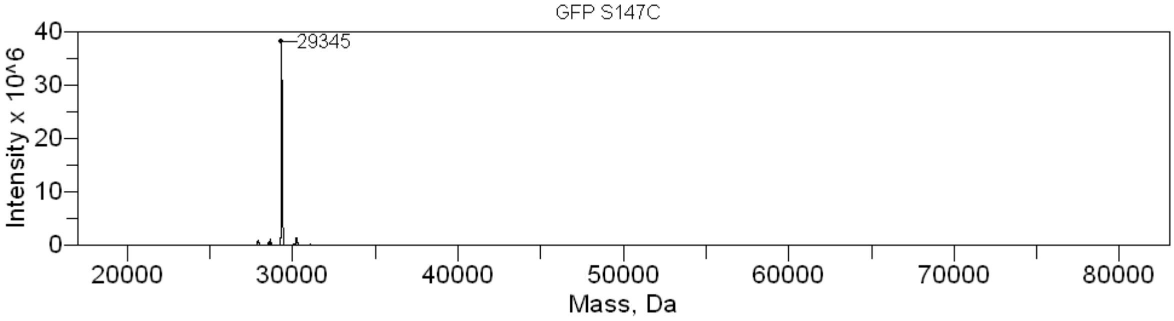

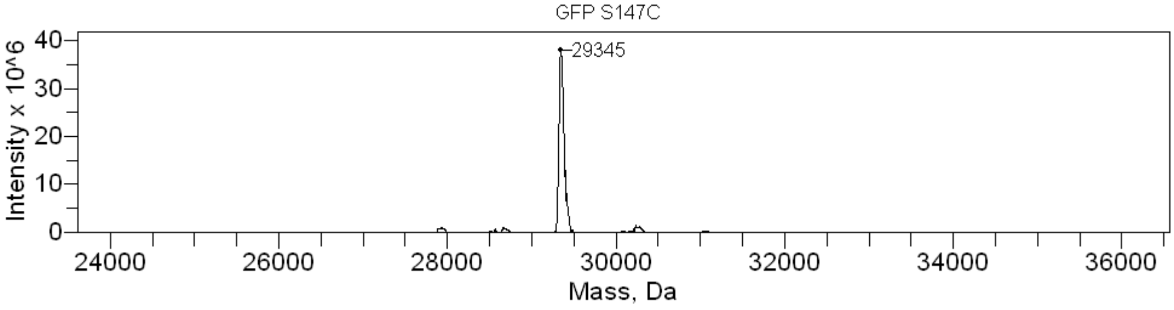

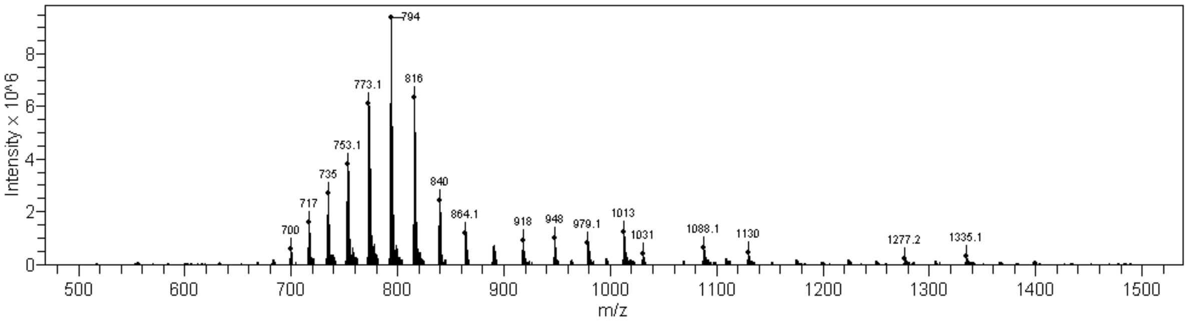

Reaction of GFP(S147C) with 2,5-dibromohexanediamide (2)

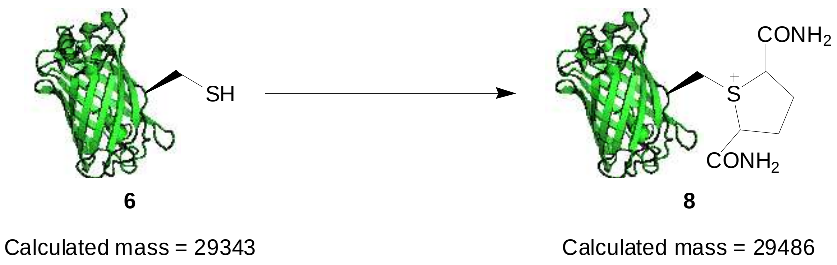

2,5-Dibromohexanediamide (**2**) (10 µL, 340 mM solution in DMF, 1000 equivalents) was added to a solution of GFP(S147C) (100 µL, 1.0 mg/mL) in sodium phosphate (100 mM, pH 8.0) at 21 °C. The mixture was vortexed for 1 s and then maintained at 21 °C for 30 min. The solid precipitates were removed by centrifugation (1 min, 12K g) and the supernatant was heated to 37 °C for 1 h. The reaction was analyzed by LCMS. Sulphonium **8** formed in >95% conversion.

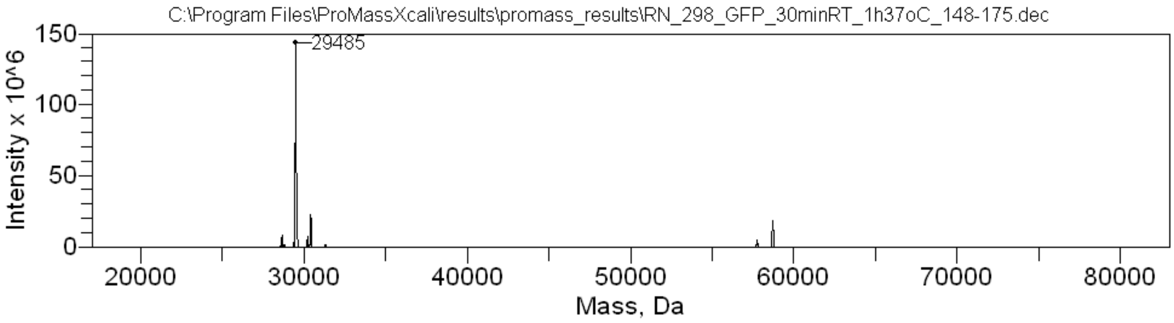

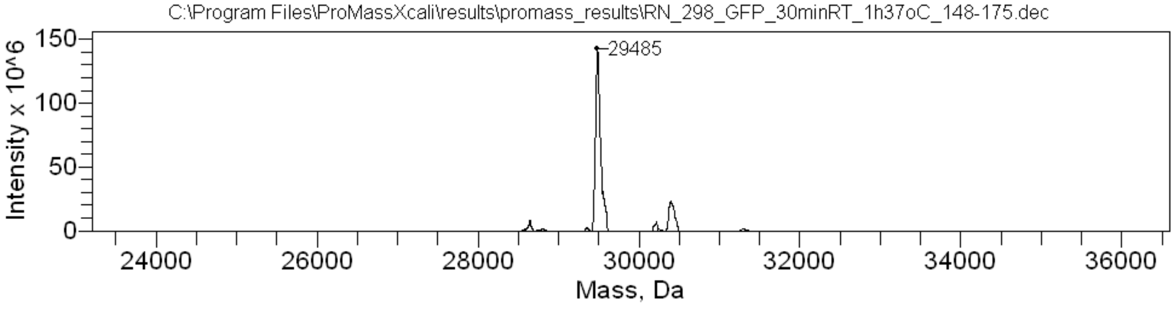

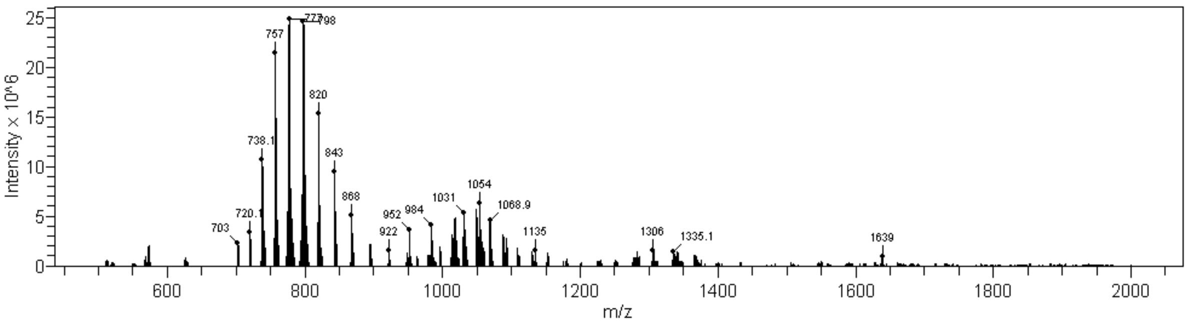

Optimization of formation of sulphonium 8

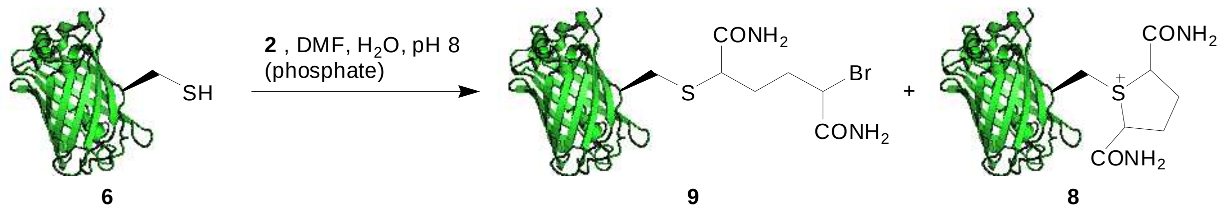

2,5-Dibromohexanediamide (**2**) (10, 25 or 50 equivalents), as a solution in DMF (** mL), was added to a solution of GFP(S147C) (100 µL, 1.0 mg/mL) in sodium phosphate (100 mM, pH 8.0) at 21 °C. The mixture was vortexed for 1 s, maintained at the required temperature (4 °C, 21 °C or 37 °C) for the prescribed time (2 h, 5 h or 20 h) and analyzed by LCMS.

<u>10 equivalents, 21 °C</u>

2 h

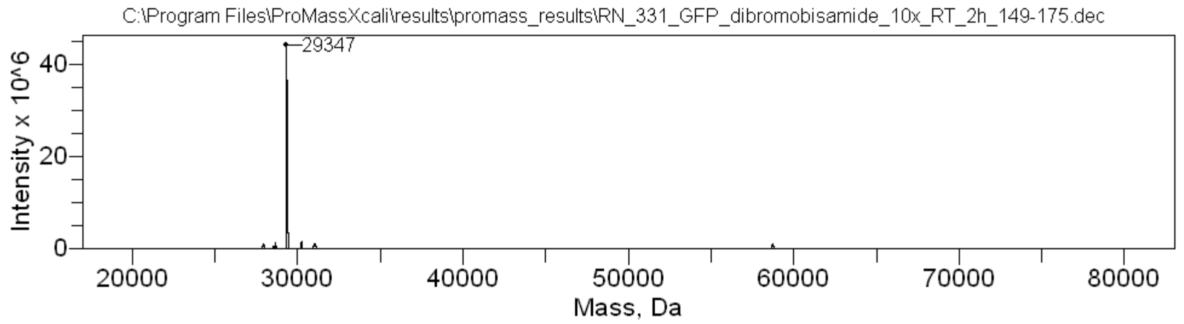

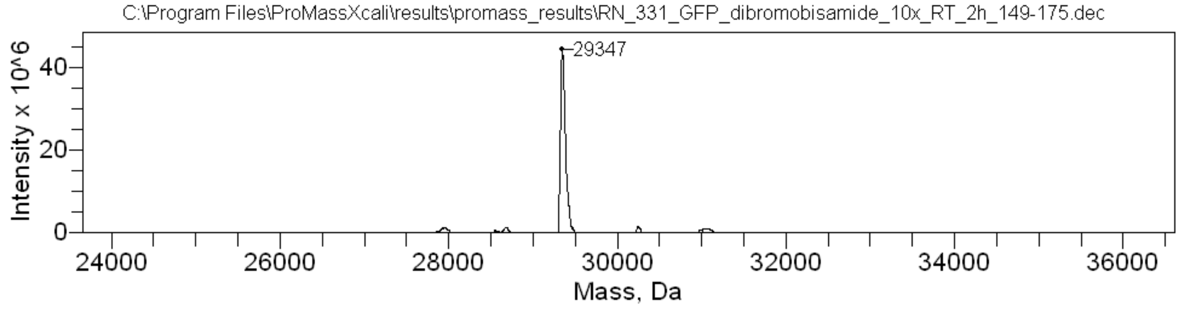

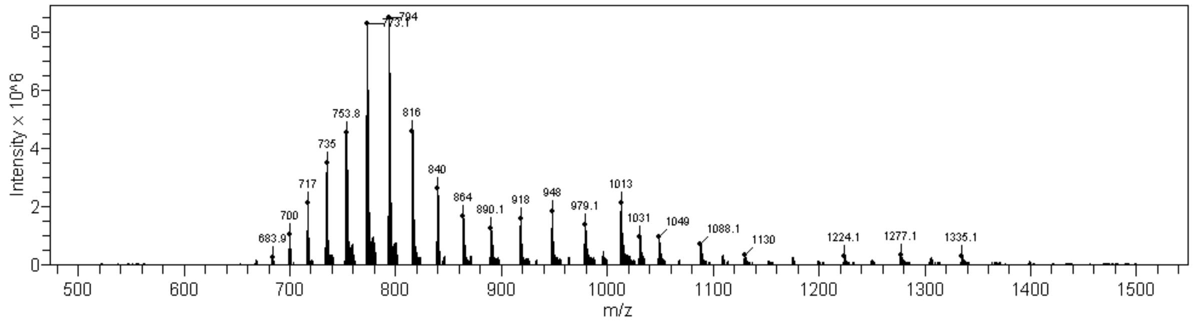

5 h

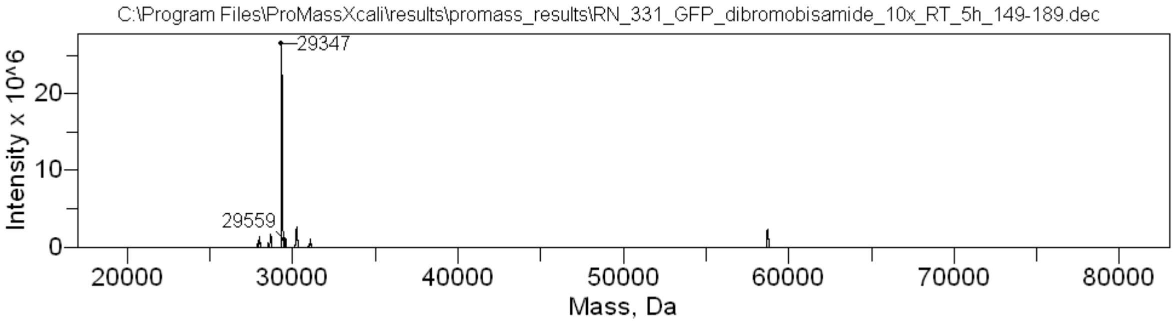
C:\Program Files\ProMassXcali\results\promass_results\RN_331_GFP_dibromobisamide_10x_RT_5h_149-189.dec
29347
29559
Intensity x 10^6
Mass, Da

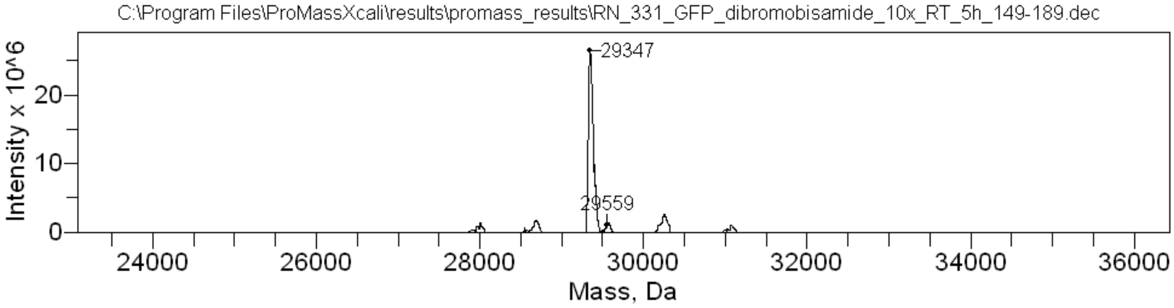
C:\Program Files\ProMassXcali\results\promass_results\RN_331_GFP_dibromobisamide_10x_RT_5h_149-189.dec
29347
29559
Intensity x 10^6
Mass, Da

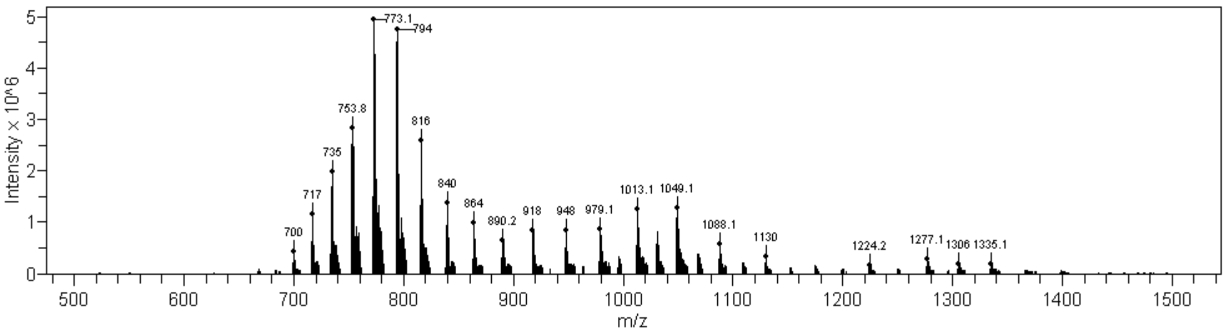
773.1
794
753.8
816
735
840
717
864
890.2
918
948
979.1
1013.1
1049.1
700
1088.1
1130
1224.2
1277.1
1306
1335.1
Intensity x 10^6
m/z

20 h

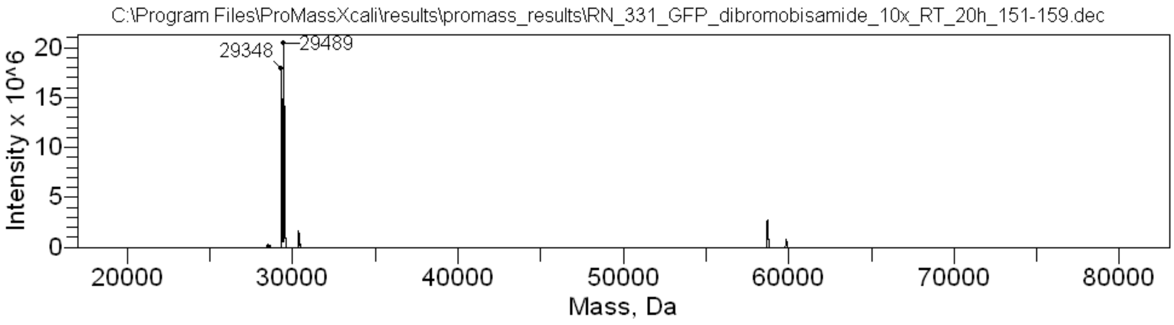
C:\Program Files\ProMassXcali\results\promass_results\RN_331_GFP_dibromobisamide_10x_RT_20h_151-159.dec
29348
29489
Intensity x 10^6
Mass, Da

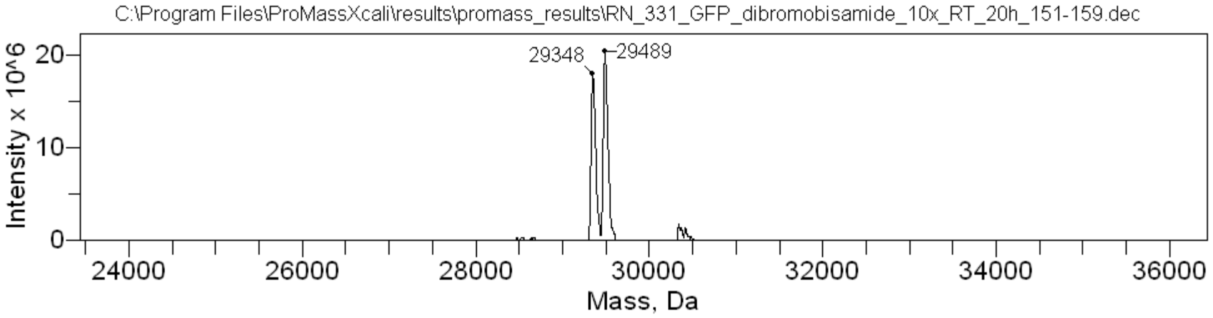
C:\Program Files\ProMassXcali\results\promass_results\RN_331_GFP_dibromobisamide_10x_RT_20h_151-159.dec
29348
29489
Intensity x 10^6
Mass, Da

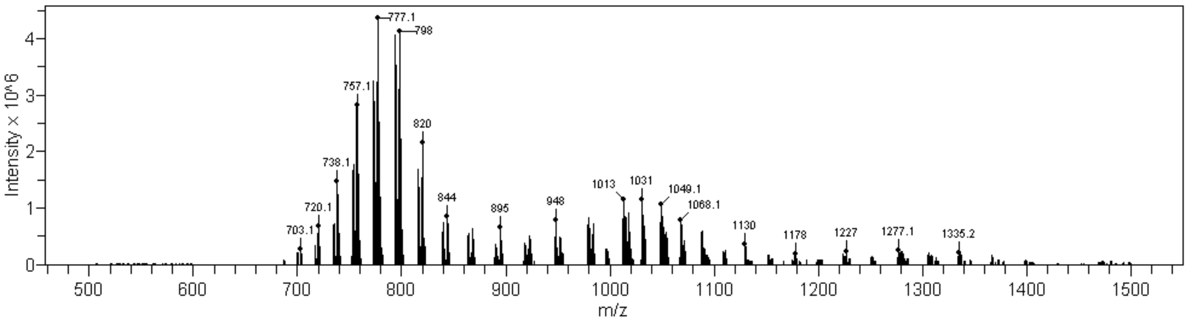

<u>25 equivalents, 21 °C</u>

2 h

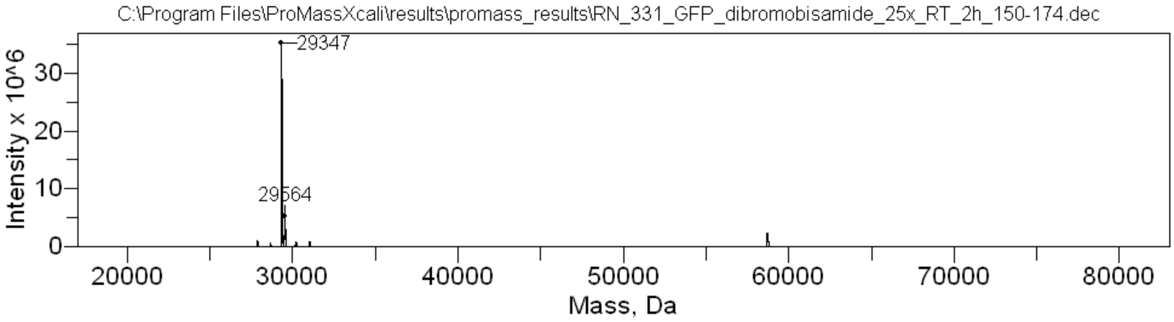

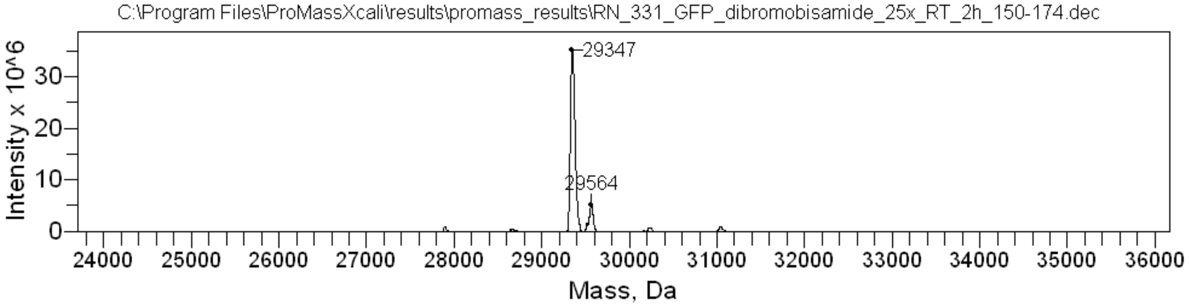

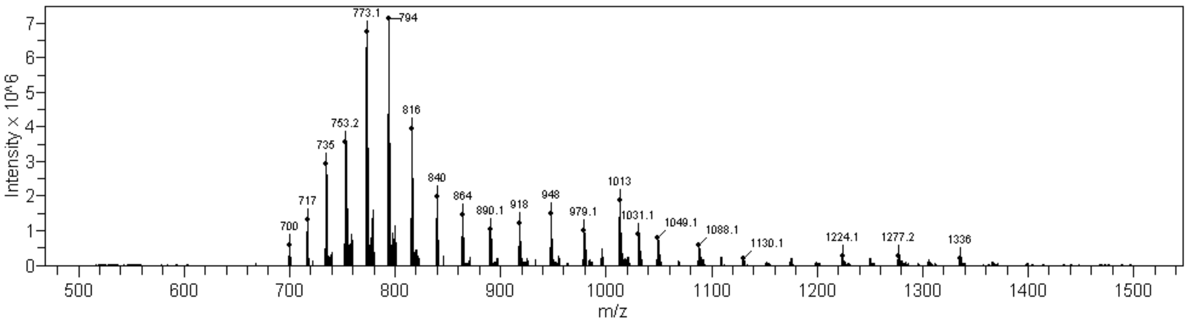

5 h

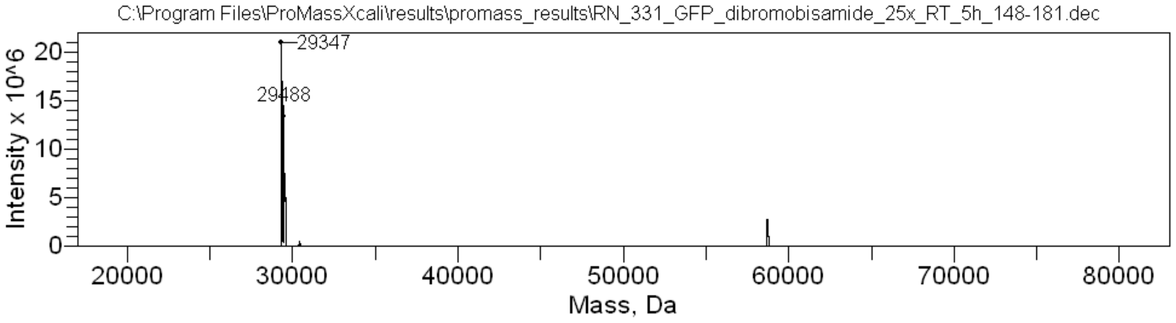
C:\Program Files\ProMassXcali\results\promass_results\RN_331_GFP_dibromobisamide_25x_RT_5h_148-181.dec
Intensity x 10^6
29347
29488
Mass, Da

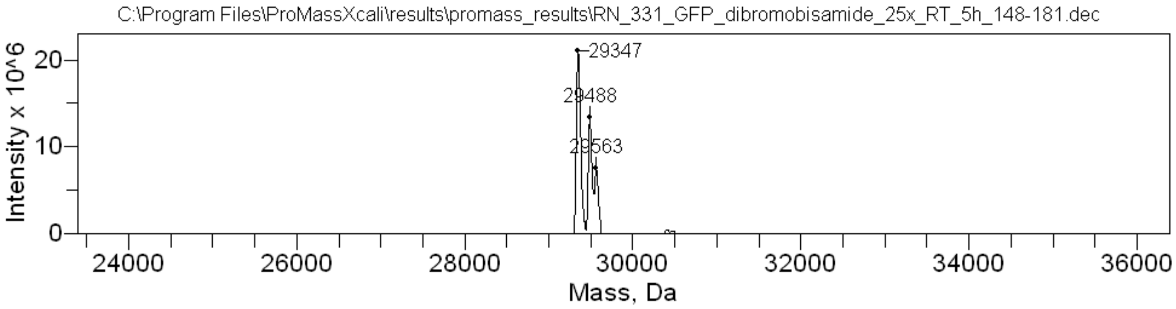
C:\Program Files\ProMassXcali\results\promass_results\RN_331_GFP_dibromobisamide_25x_RT_5h_148-181.dec
Intensity x 10^6
29347
29488
29563
Mass, Da

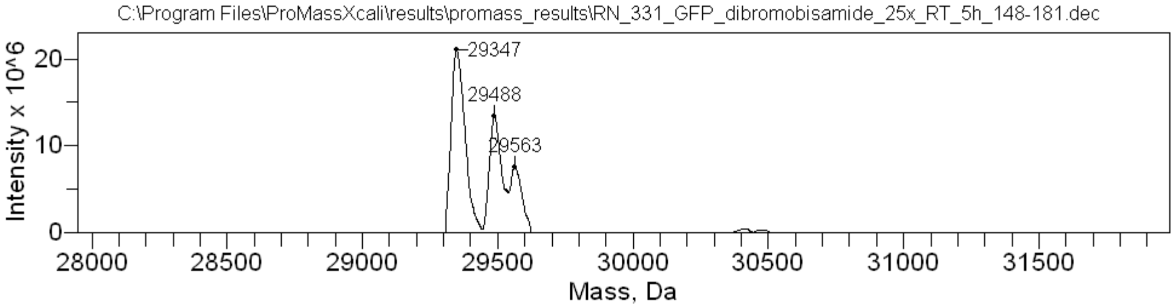
C:\Program Files\ProMassXcali\results\promass_results\RN_331_GFP_dibromobisamide_25x_RT_5h_148-181.dec
Intensity x 10^6
29347
29488
29563
Mass, Da

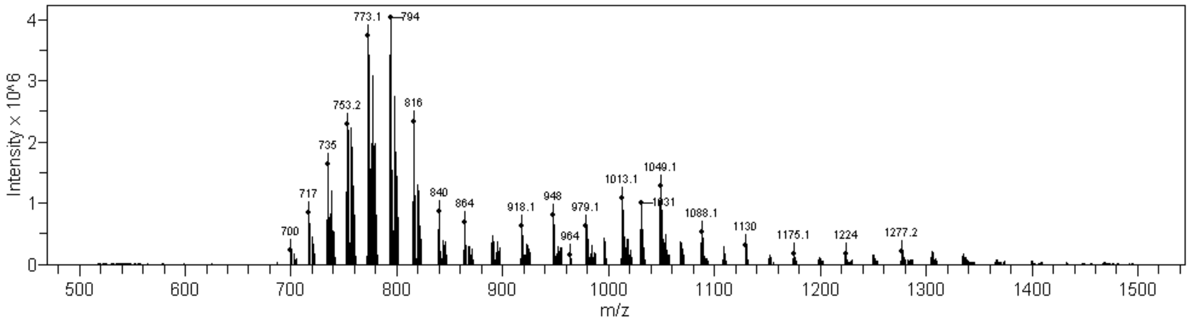
Intensity x 10^6
773.1
794
753.2
816
735
717
700
840
864
918.1
948
964
979.1
1013.1
1049.1
1031
1088.1
1130
1175.1
1224
1277.2
m/z

20 h

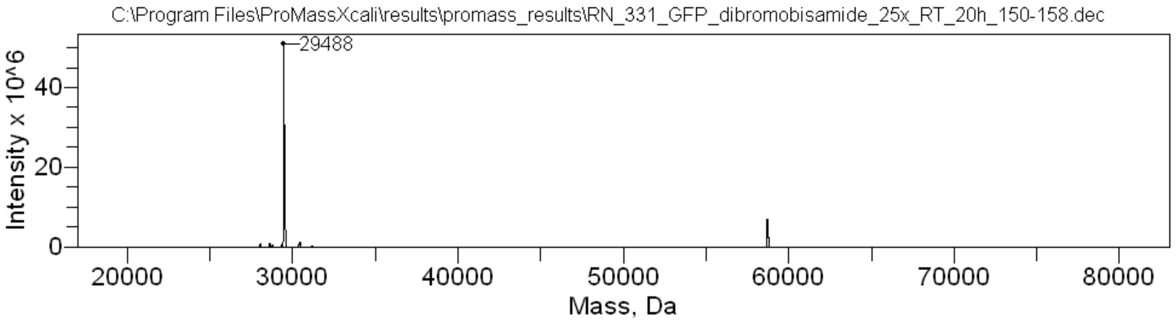
C:\Program Files\ProMassXcali\results\promass_results\RN_331_GFP_dibromobisamide_25x_RT_20h_150-158.dec
Intensity x 10^6
29488
Mass, Da

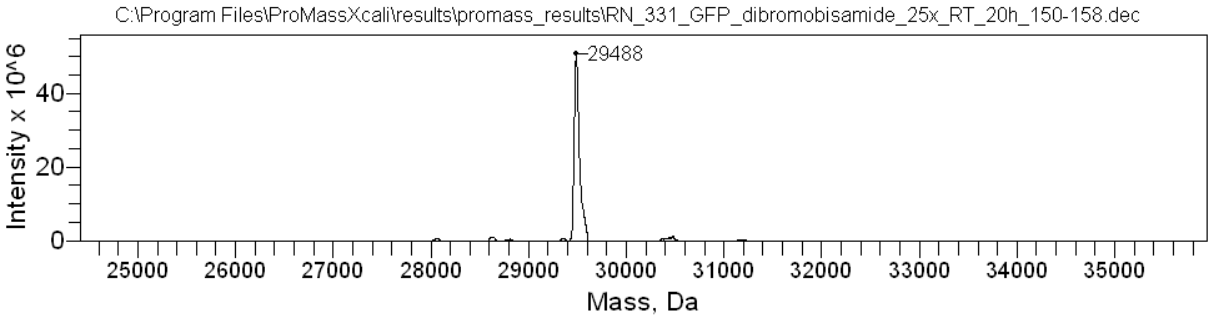

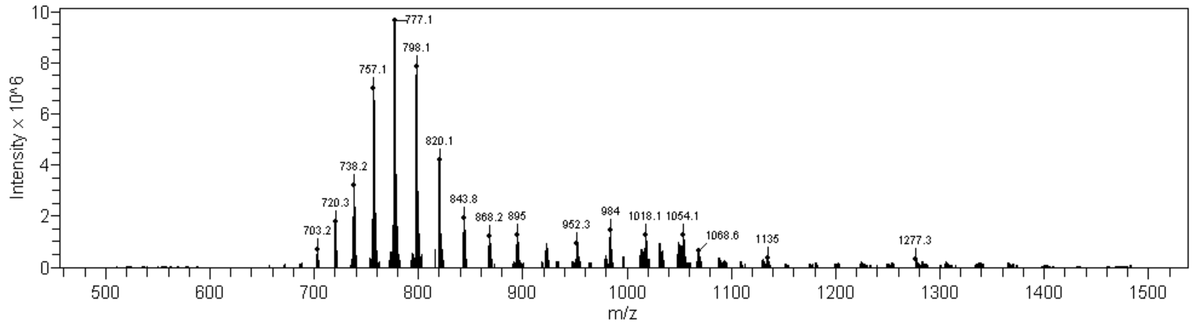

<u>50 equivalents, 21 °C</u>

2 h

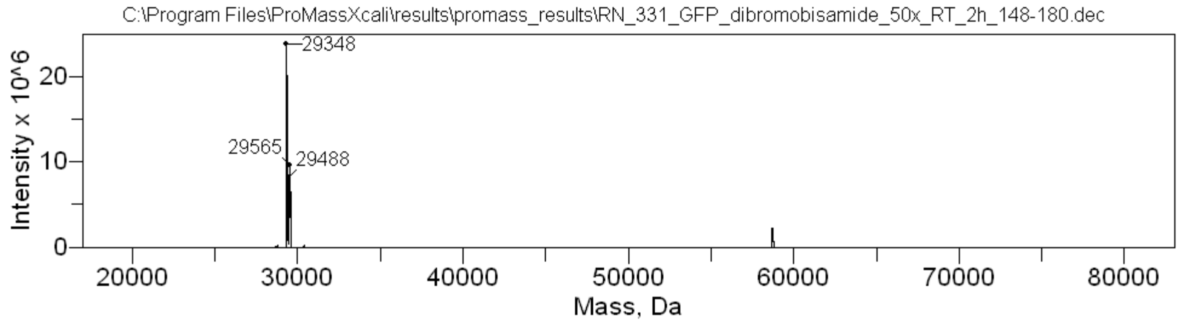

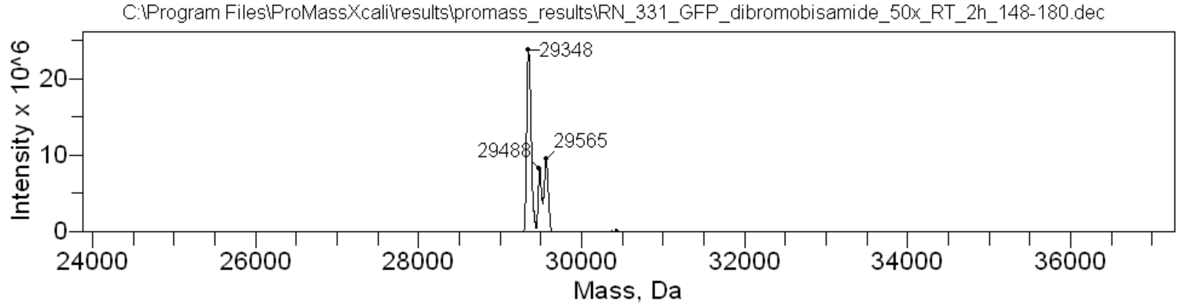

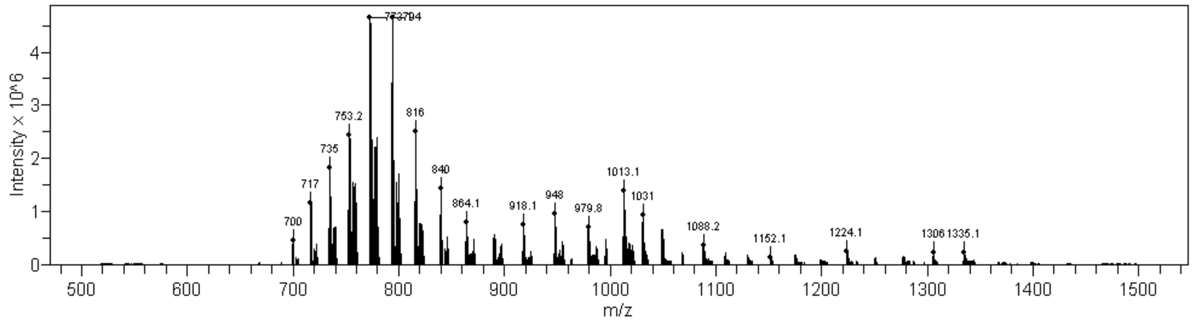

5 h

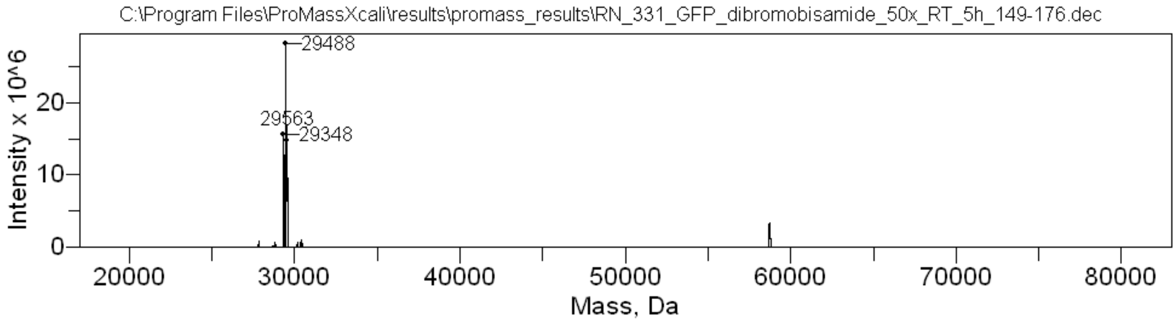
C:\Program Files\ProMassXcali\results\promass_results\RN_331_GFP_dibromobisamide_50x_RT_5h_149-176.dec
29488
29563
29348
Intensity x 10^6
Mass, Da

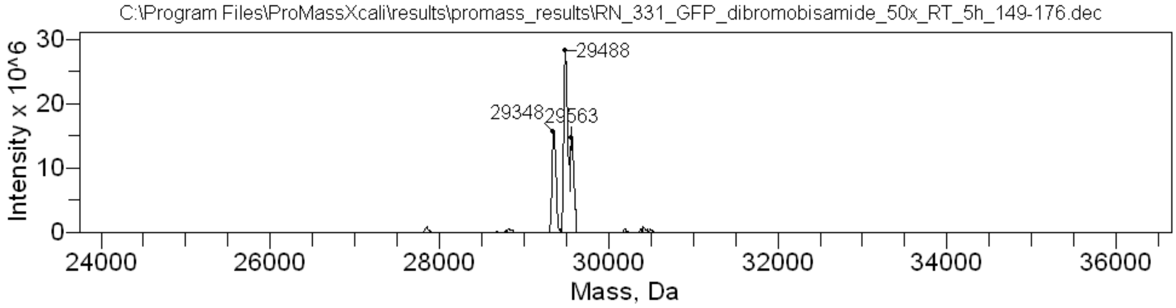
C:\Program Files\ProMassXcali\results\promass_results\RN_331_GFP_dibromobisamide_50x_RT_5h_149-176.dec
29488
29348
29563
Intensity x 10^6
Mass, Da

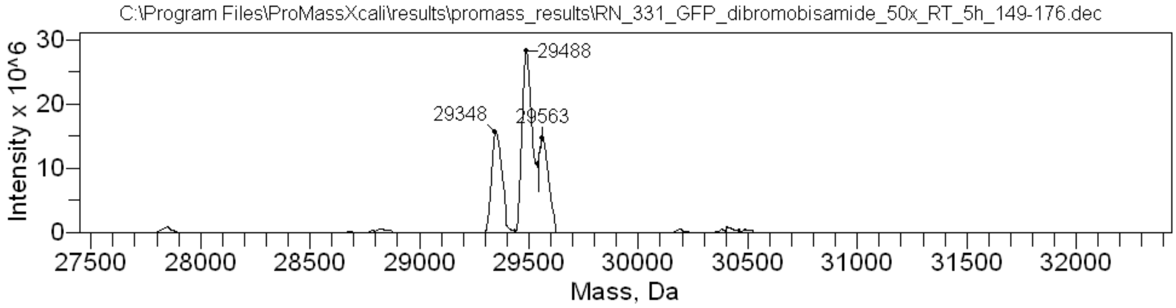
C:\Program Files\ProMassXcali\results\promass_results\RN_331_GFP_dibromobisamide_50x_RT_5h_149-176.dec
29488
29348
29563
Intensity x 10^6
Mass, Da

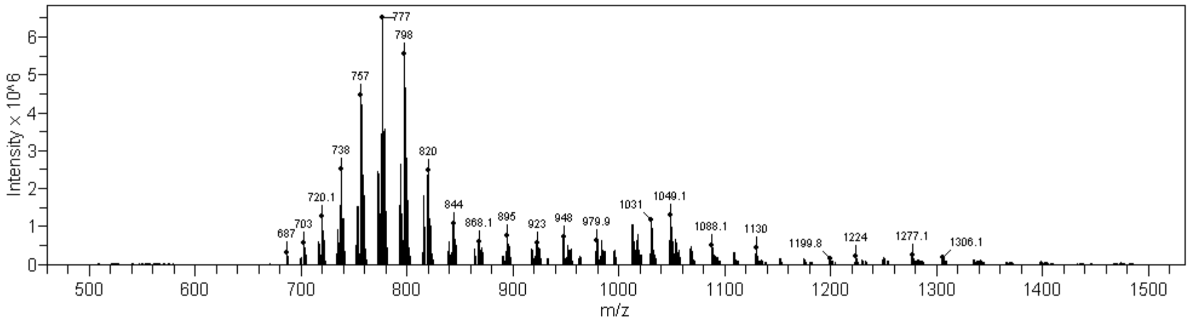
777
798
757
738
820
720.1
703
687
844
868.1
895
923
948
979.9
1031
1049.1
1088.1
1130
1199.8
1224
1277.1
1306.1
Intensity x 10^6
m/z

20 h

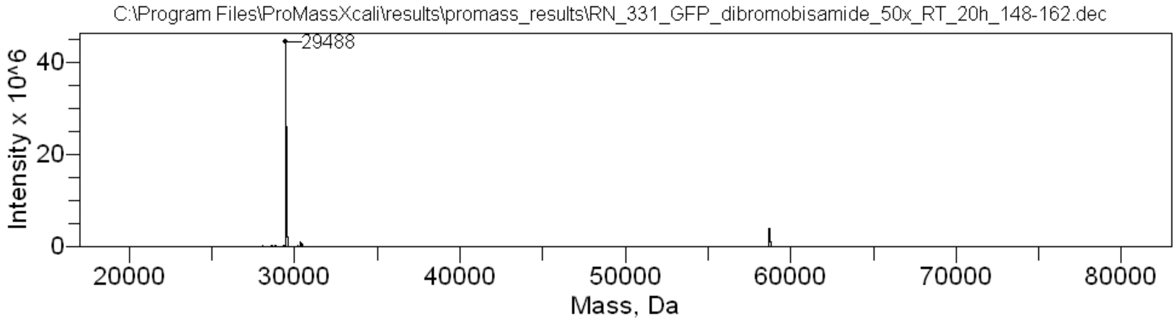
C:\Program Files\ProMassXcali\results\promass_results\RN_331_GFP_dibromobisamide_50x_RT_20h_148-162.dec
29488
Intensity x 10^6
Mass, Da

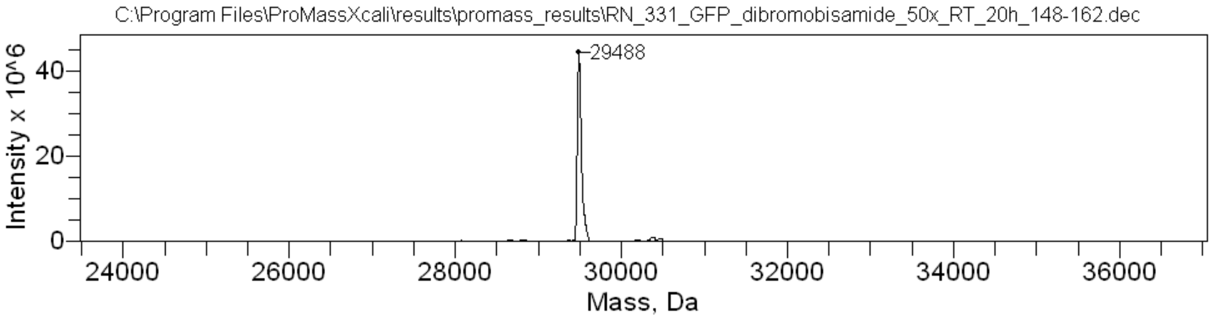

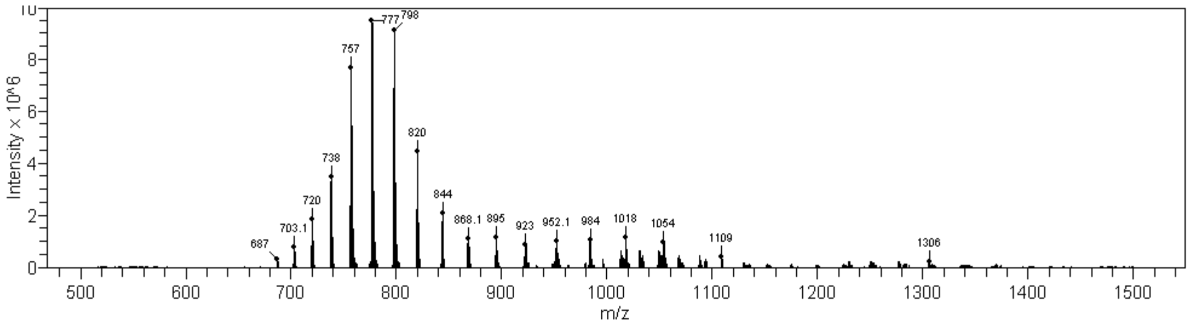

<u>10 equivalents, 4 °C, 20 h</u>

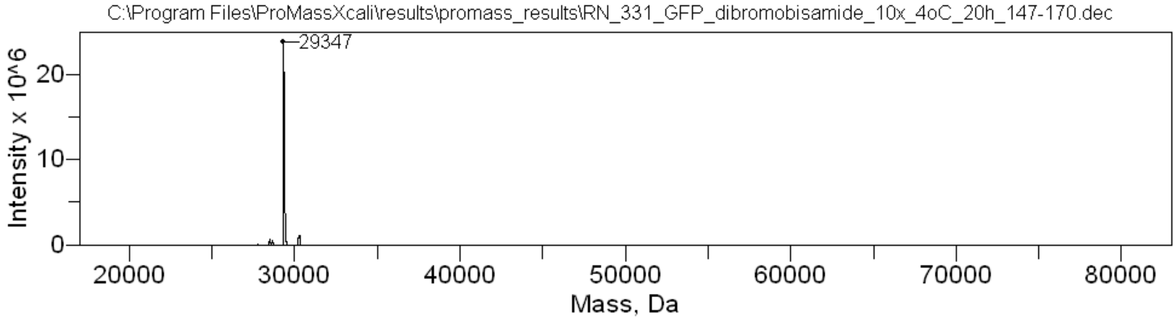

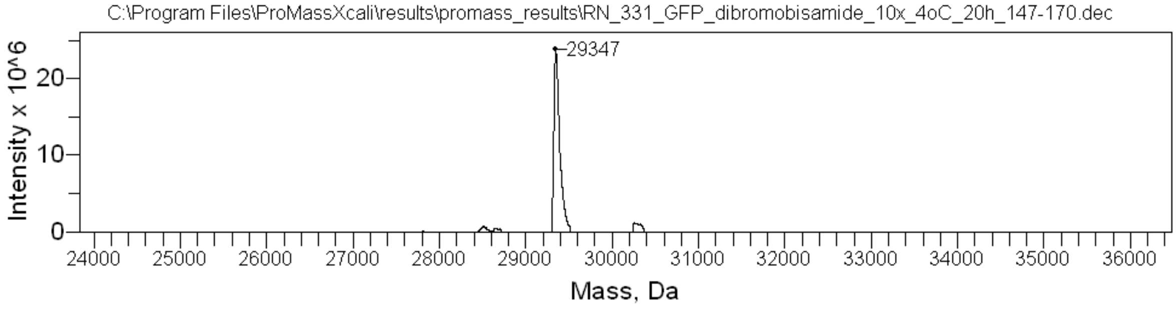

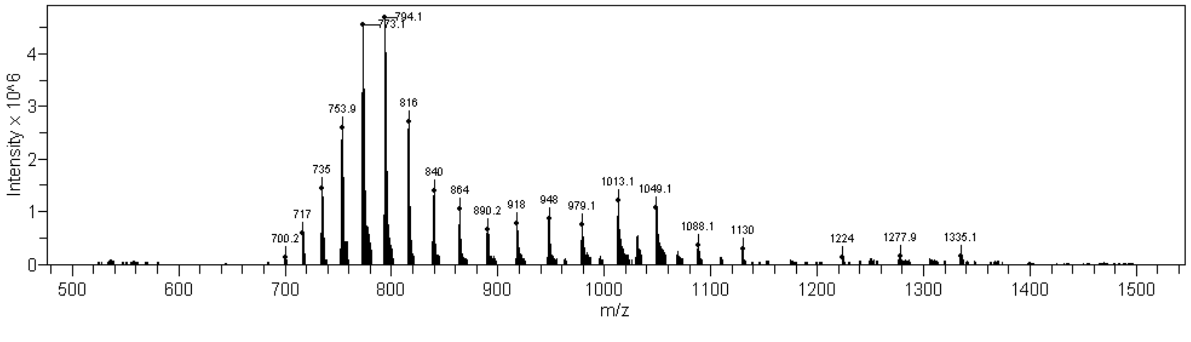

25 equivalents, 4 °C, 20 h

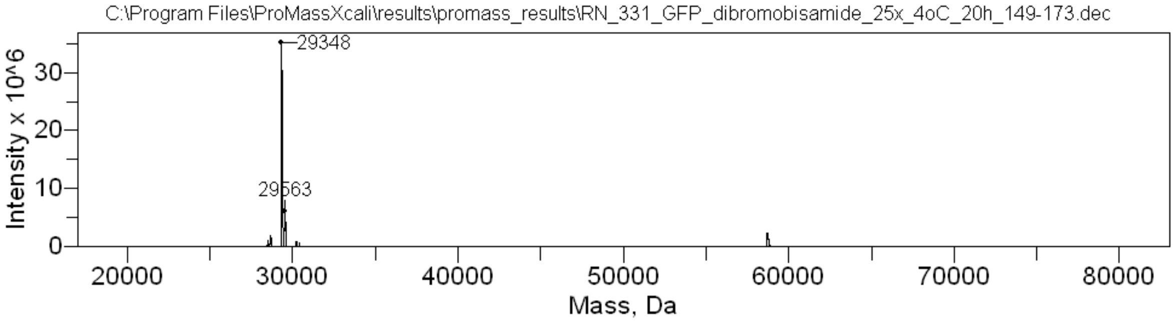

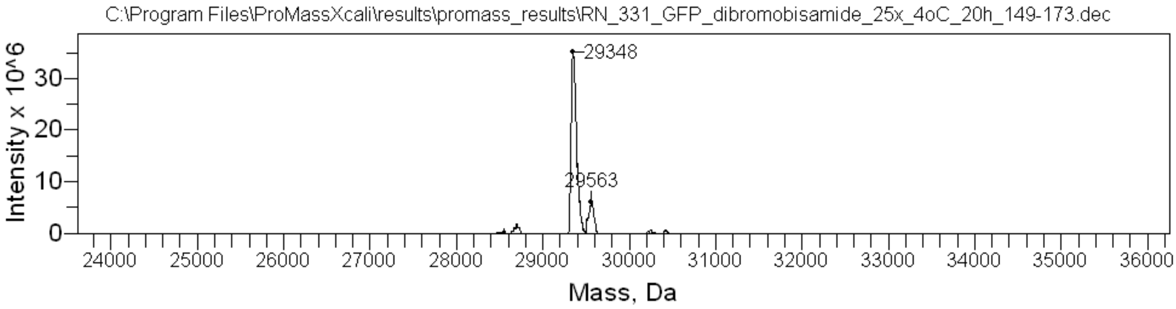

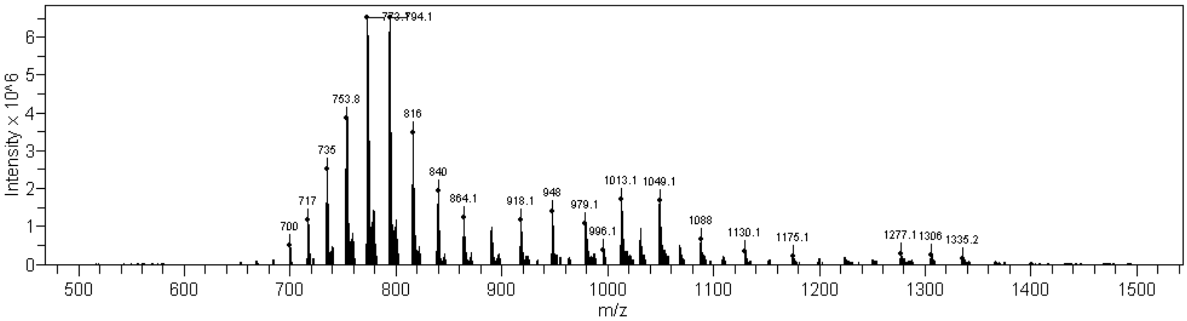

50 equivalents, 4 °C, 20 h

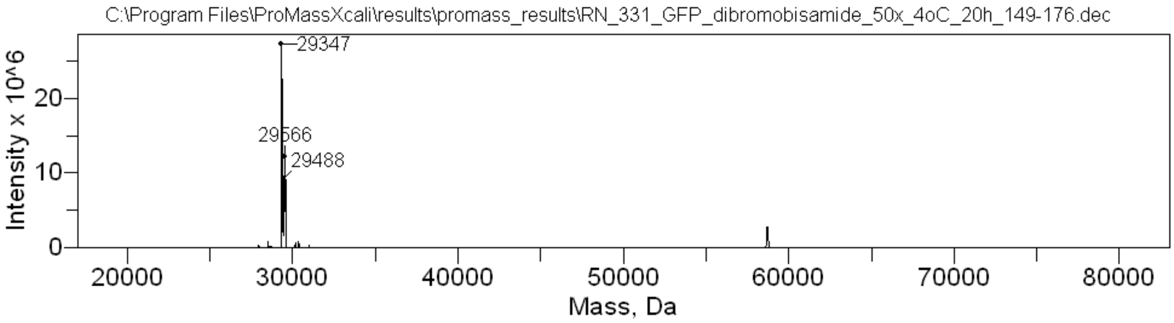

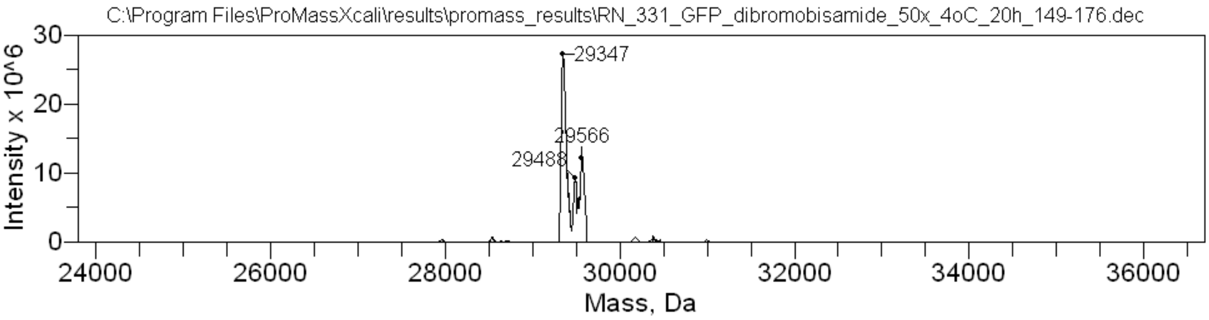

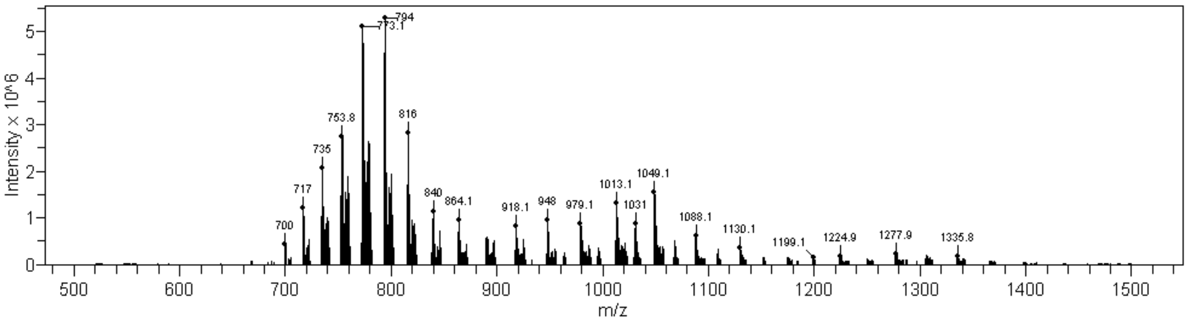

<u>50 equivalents, 37 °C, 2 h</u>

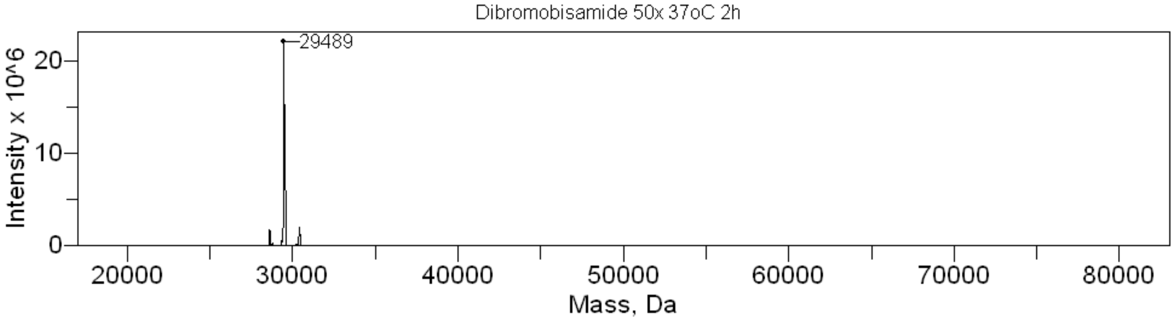

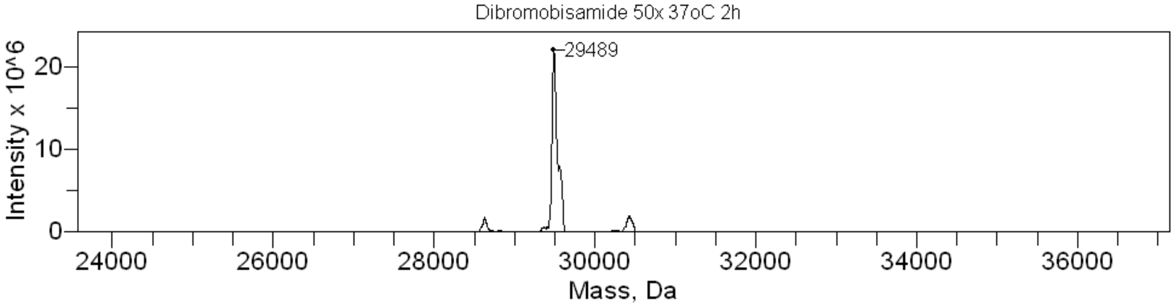

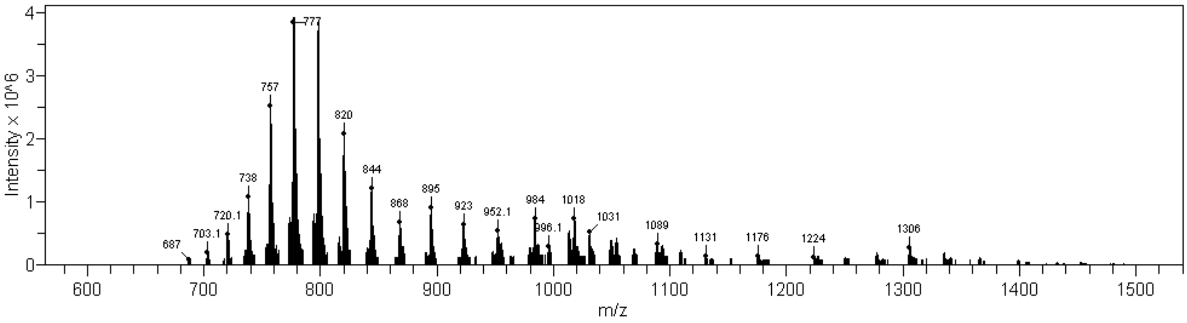

Synthesis of sulphonium 8

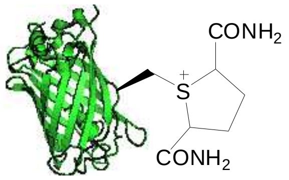

8

Calculated mass = 29486

2,5-Dibromohexanediamide (**2**) (17 mM solution in DMF, 50 equivalents) was added to a solution of GFP(S147C) (1.0 mg/mL) in sodium phosphate (100 mM, pH 8.0) at 21 °C. The mixture was vortexed for 1 s and then maintained at 21 °C for 20 h. Analysis by LCMS indicated complete formation of sulphonium **8**. Excess 2,5-dibromohexanediamide (**2**) was removed by repeated diafiltration into fresh buffer (100mM sodium phosphate, pH 8.0) using VivaSpin sample concentrators (GE Healthcare, 10,000 MWCO) and analyzed by LCMS.

The solution of sulfonium **8** sufficiently stable to be at 4 °C for 1 month

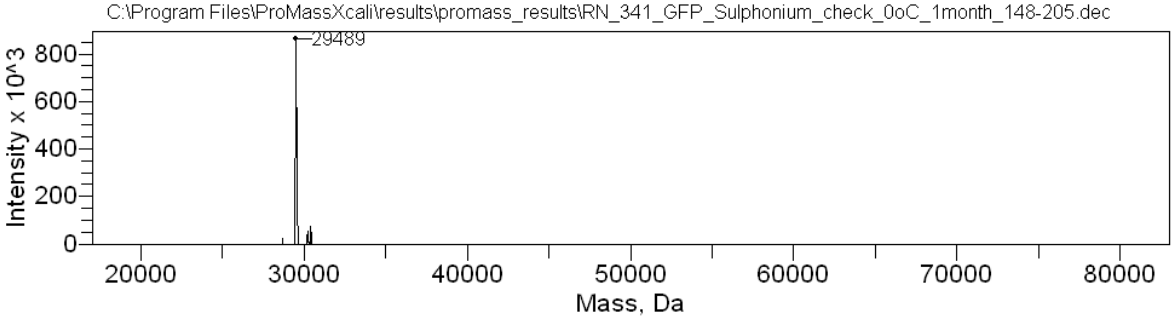

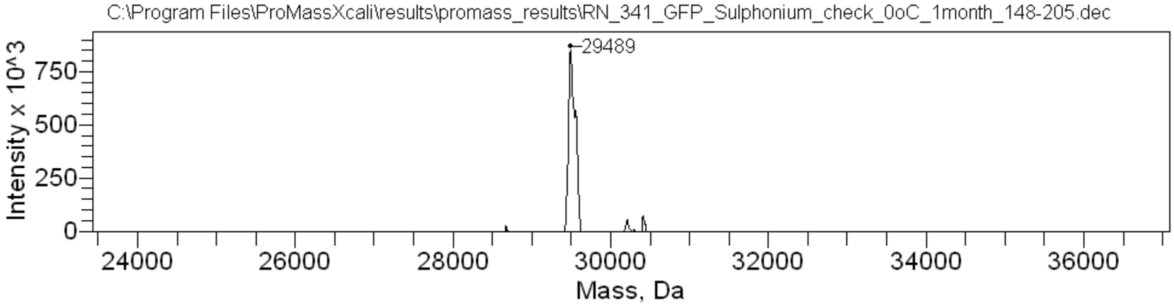

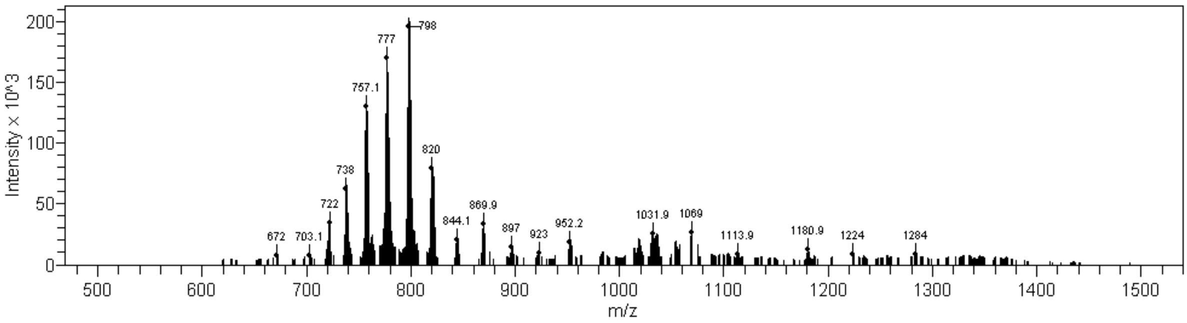

Reaction of GFP(S147C) 6 with N-methylbromomaleimide

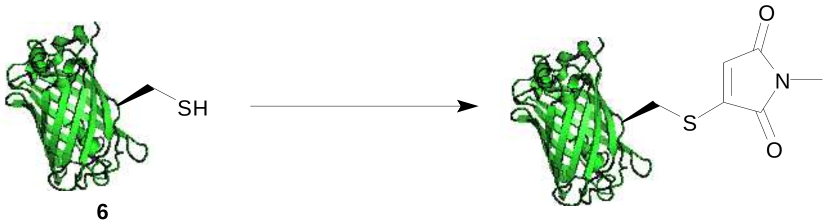

6

Calculated mass = 29343 Calculated mass = 29454

N-Methylbromomaleimide (5 µL, 6.8 mM solution in DMF, 1 equivalent) was added to a solution of GFP(S147C) **6** (100 µL, 1.0 mg/mL) in sodium phosphate (100 mM, pH 8.0) at 21 °C. The mixture was vortexed for 1 s, maintained at 21 °C for 1 h and analysed by LCMS.

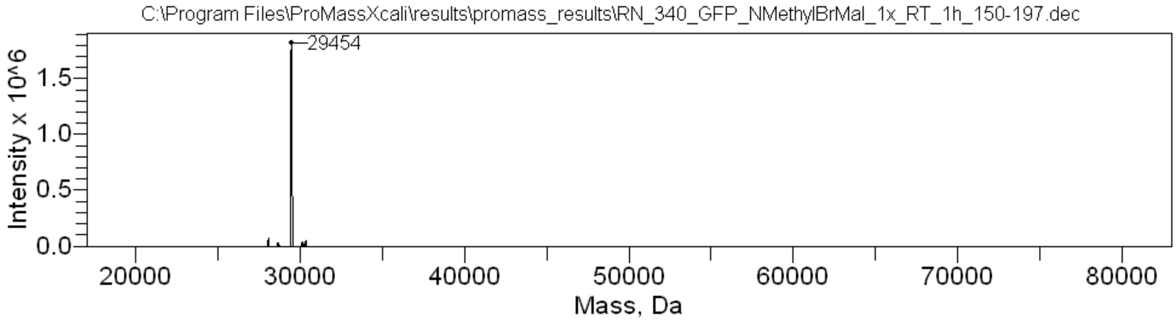

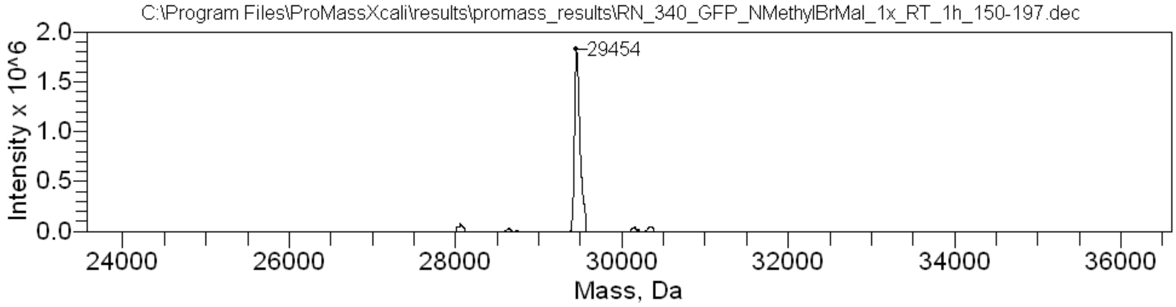

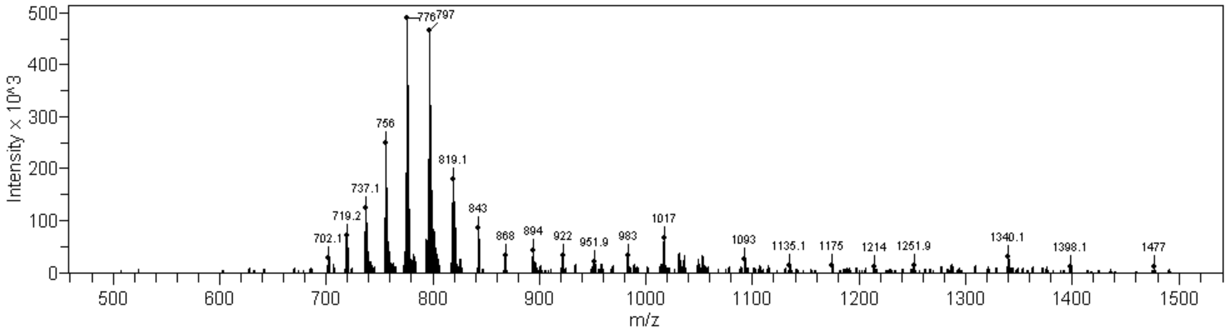

Reaction of sulphonium 8 with N-methylbromomaleimide

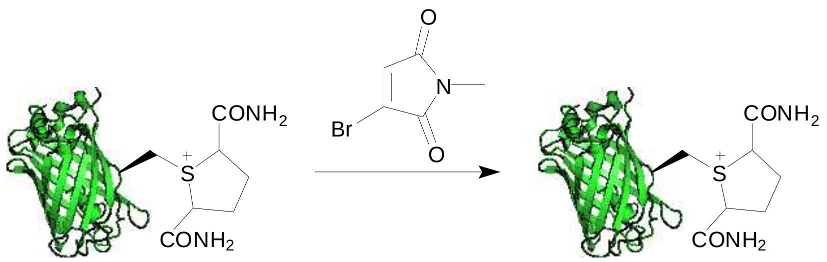

8   8

Calculated mass = 29486   Calculated mass = 29486

N-methylbromomaleimide (5 µL, 6.8 mM solution in DMF, 1 equivalent) was added to a solution of sulfonium **8** (100 µL, 1.0 mg/mL) in sodium phosphate (100 mM, pH 8.0) at 21 °C. The mixture was vortexed for 1 s, maintained at 21 °C for 1 h and analysed by LCMS.

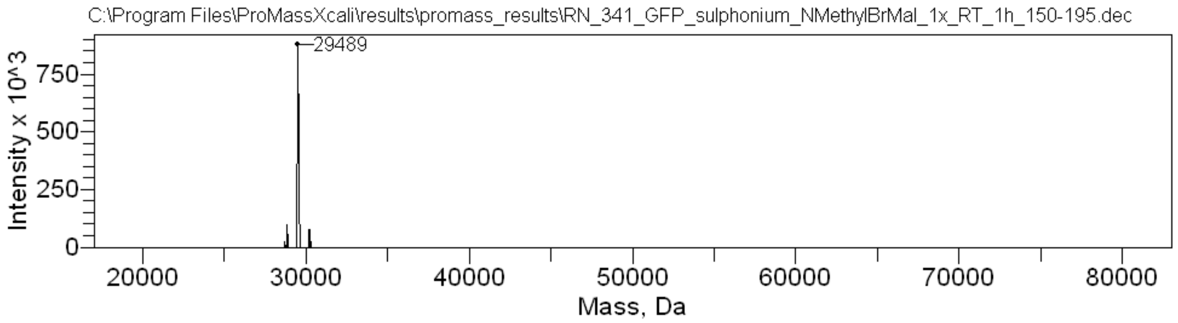

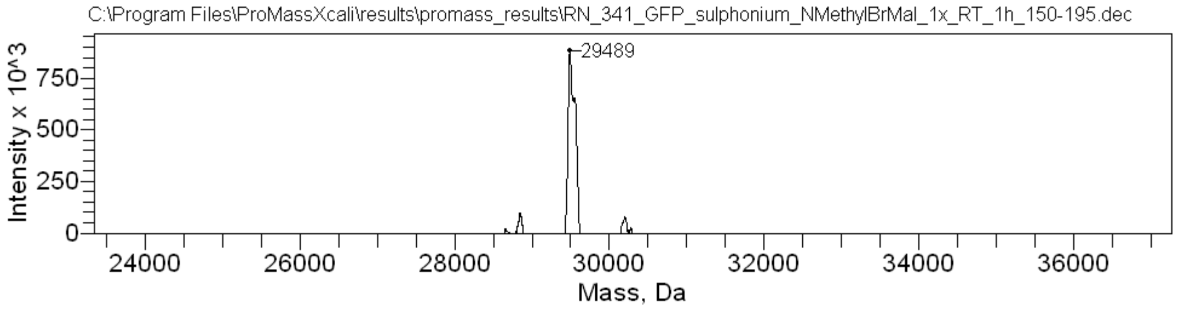

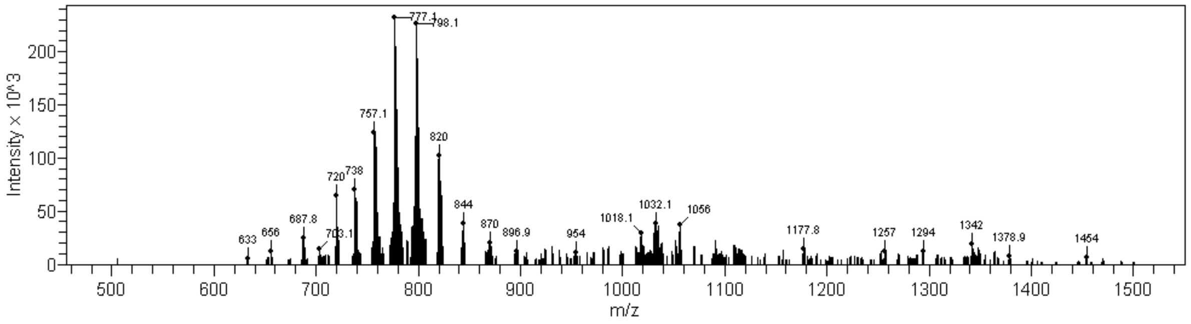

Reaction of sulphonium 8 with β-mercaptoethanol

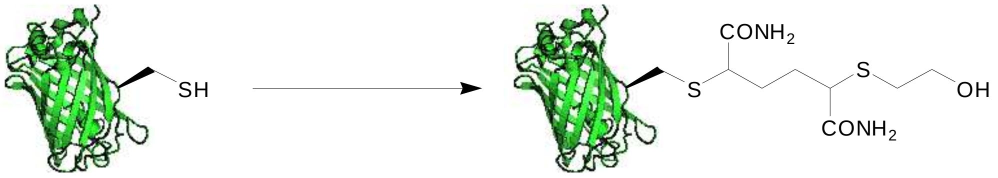

6

Calculated mass = 29343

10a

Calculated mass = 29564

β-Mercaptoethanol (5 μL, 680 mM solution in H_2O, 1000 equivalents) was added to a solution of sulphonium **8** (formed from reaction of GFP(S147C) **6** with 2,5-dibromohexanediamide (**2**) (1000 equivalents)). The mixture was vortexed for 1 s, maintained at 37 °C for 2.5 h and analysed by LCMS

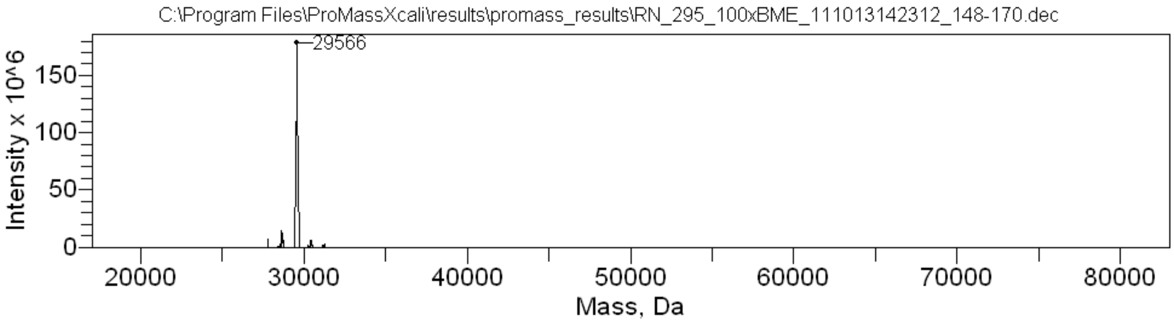

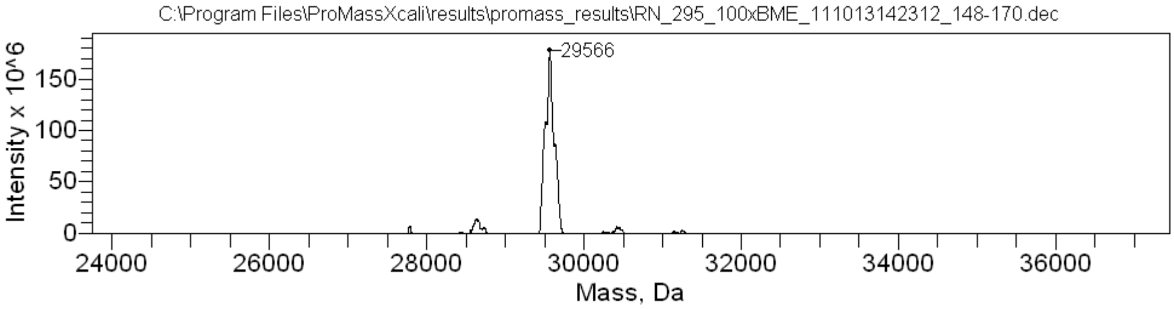

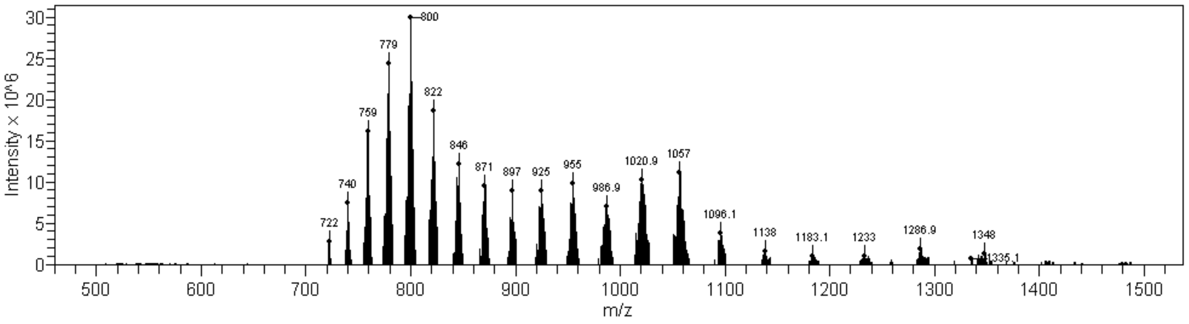

Reaction of sulphonium 8 with thioglusose

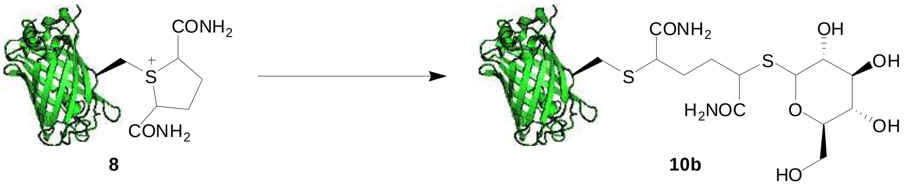

Calculated mass = 29486

Calculated mass = 29681

Thioglucose (5 μL in H_2O, 10 or 100 equivalents) was added to a solution of sulfonium **8** (100 μL, 1.0 mg/mL) in sodium phosphate (100 mM, pH 8.0) at 21 °C. The mixture was vortexed for 1 s, maintained at the required temperature (21 or 37 °C) for 5 h and analysed by LCMS

10 equivalents, 37 °C, 5 h

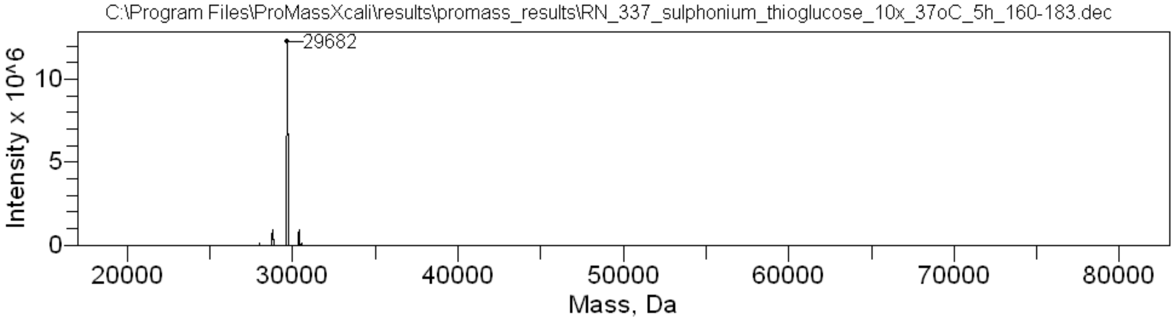

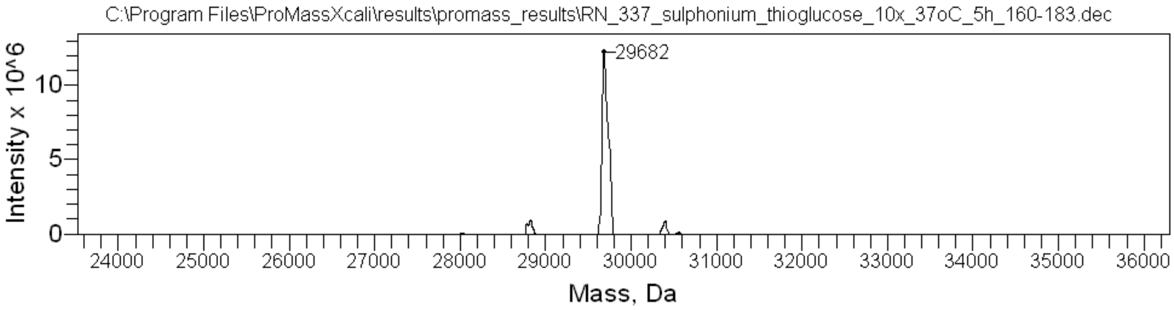

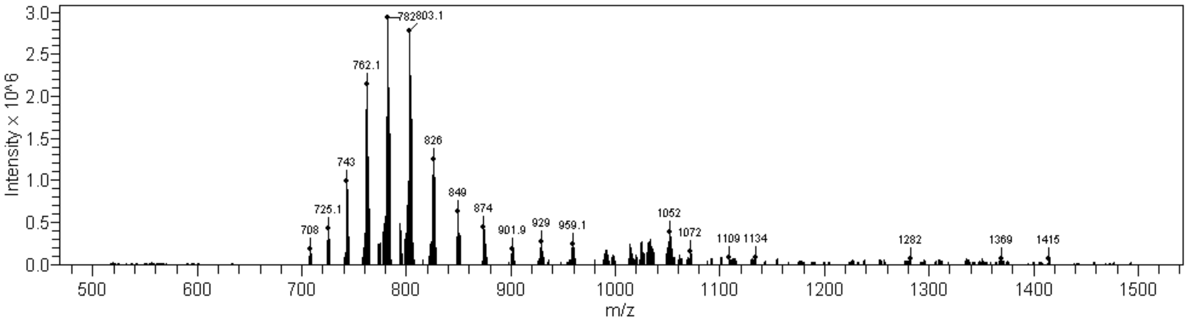

100 equivalents, 21 °C, 5 h

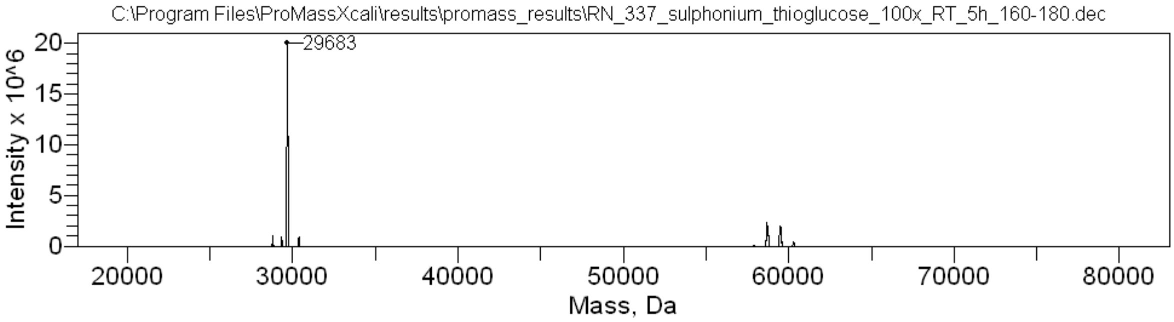

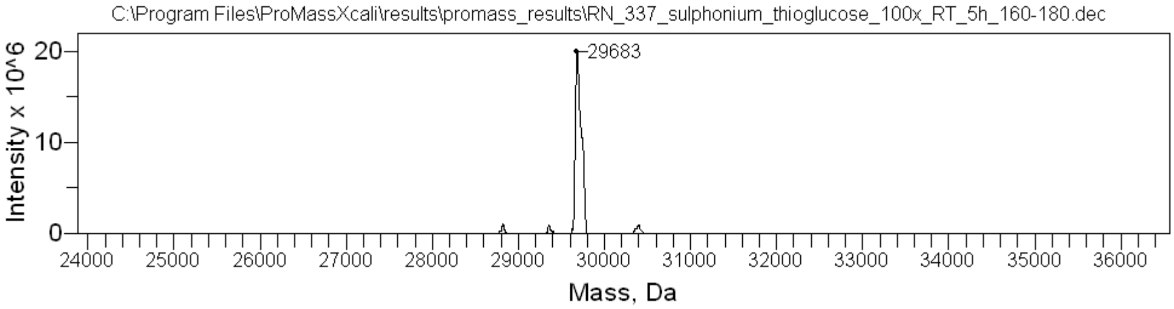

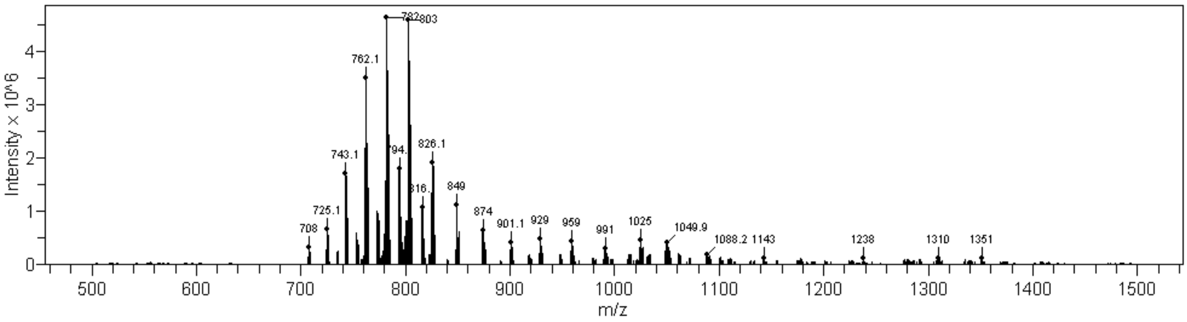

Reaction of sulphonium 8 with glutathione

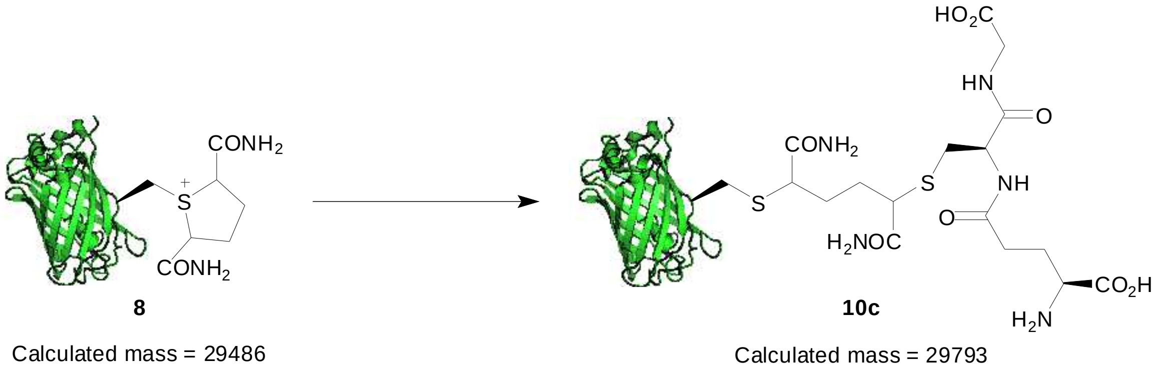

Glutathione (5 μL in H_2O, 10, 100 or 200 equivalents) was added to a solution of sulfonium **8** (100 μL, 1.0 mg/mL) in sodium phosphate (100 mM, pH 8.0) at 21 °C. The mixture was vortexed for 1 s, maintained at the required temperature (21 or 37 °C) for the prescribed time (2 h or 5 h) and analysed by LCMS.

<u>10 equivalents, 37 °C, 5 h</u>

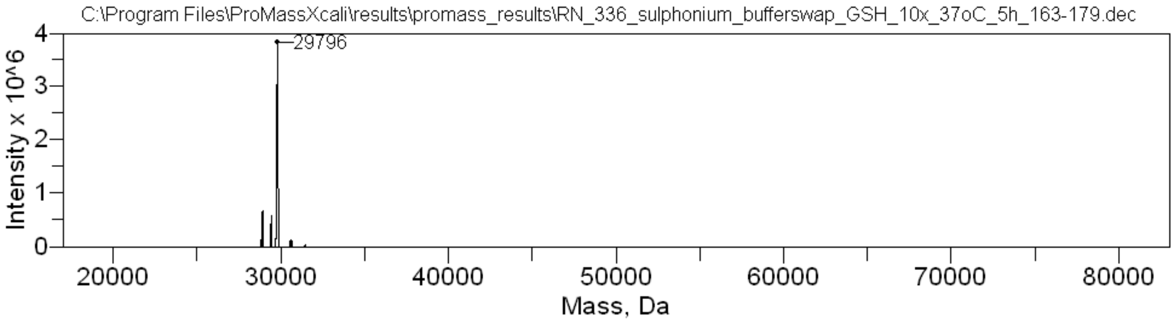

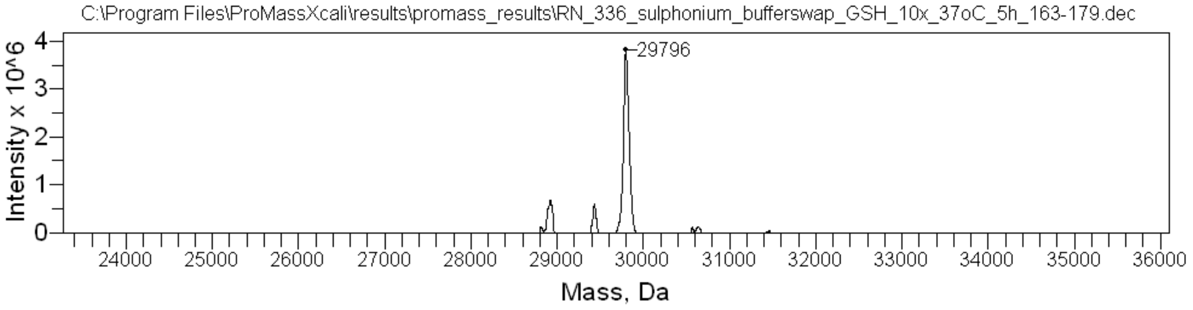

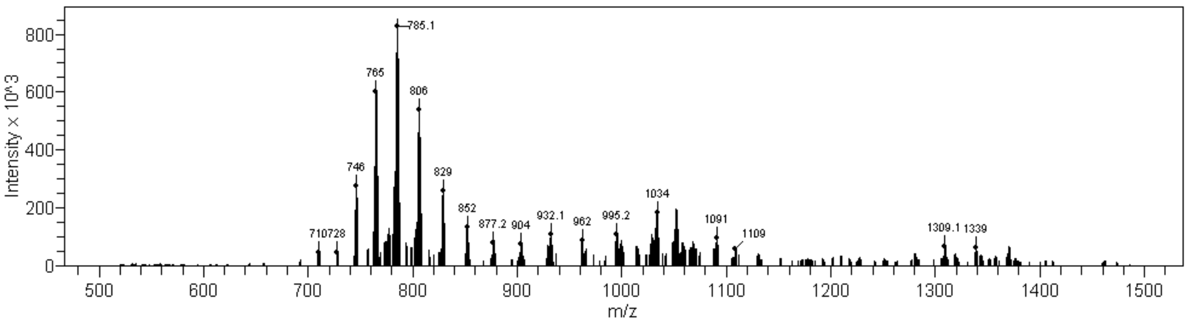

100 equivalents, 21 °C, 5 h

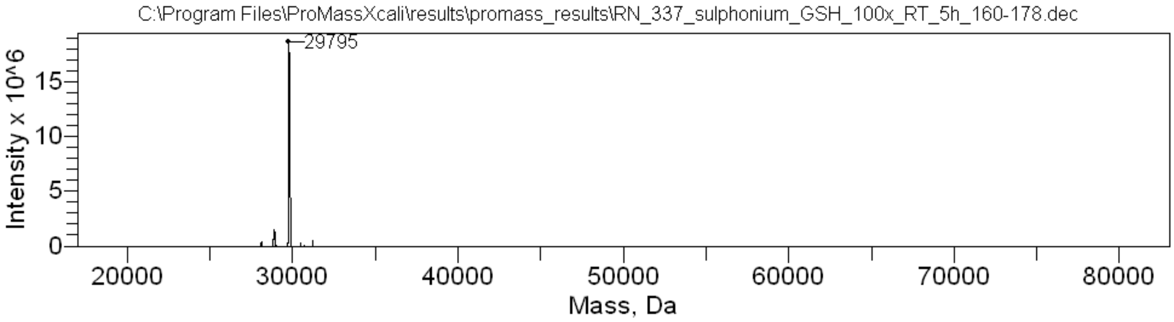

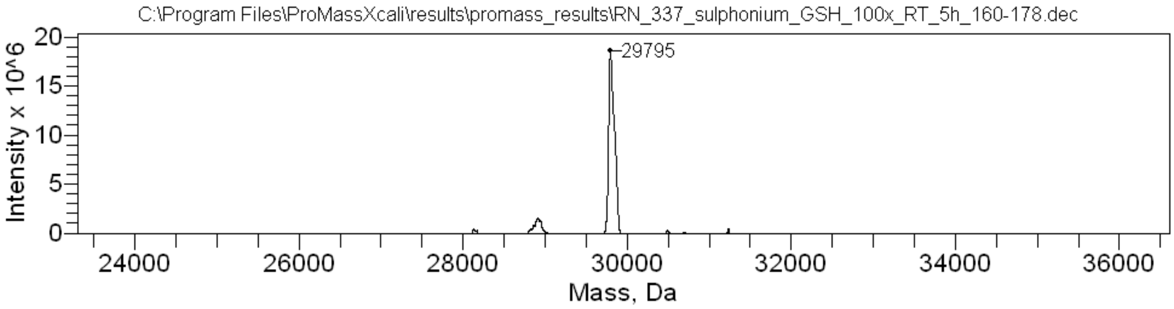

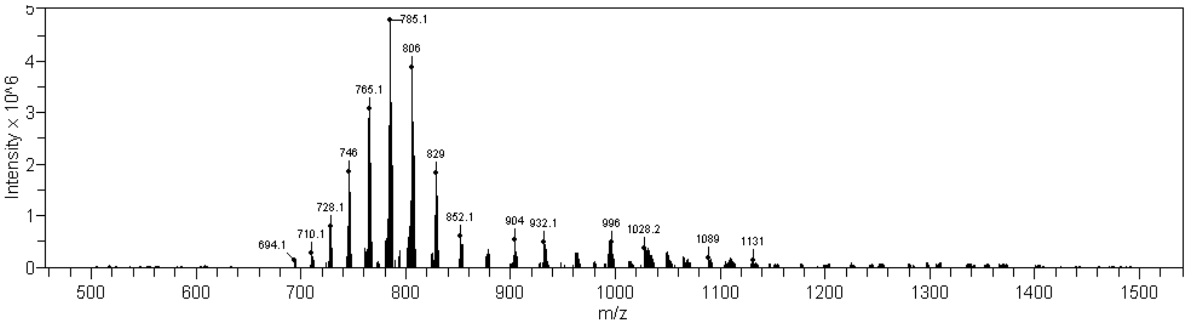

200 equivalents, 37 °C, 2 h

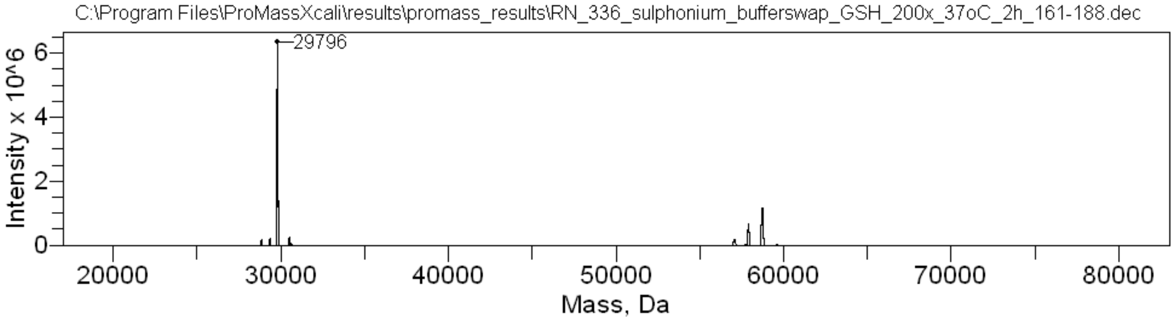

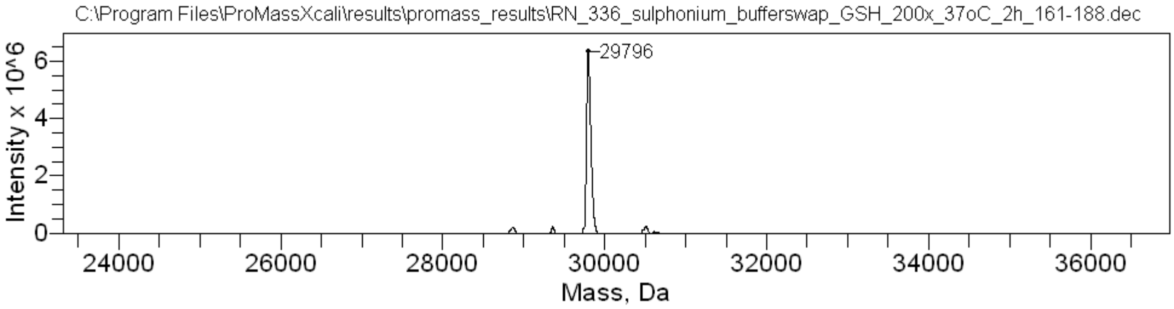

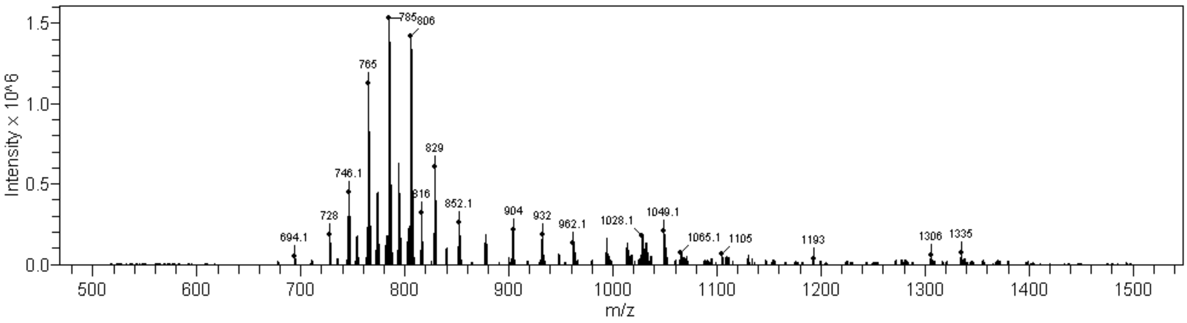

Intensity x 10^6
m/z
785
806
765
829
746.1
816
852.1
728
904
932
962.1
1028.1
1049.1
694.1
1065.1
1105
1193
1306
1335

Reaction of sulphonium 8 with benzeneselenol

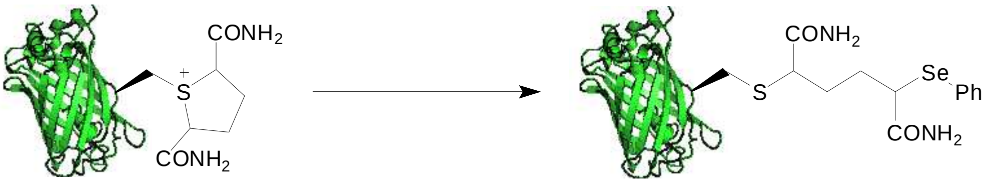

8 Calculated mass = 29486

10d Calculated mass = 29643

Benzeneselenol (5 µL, 68 mM solution in DMF, 100 equivalents) was added to a solution of sulfonium **8** (100 µL, 1.0 mg/mL) in sodium phosphate (100 mM, pH 8.0) at 21 °C. The mixture was vortexed for 1s, maintained at the 37 °C for 5 h and analysed by LCMS.

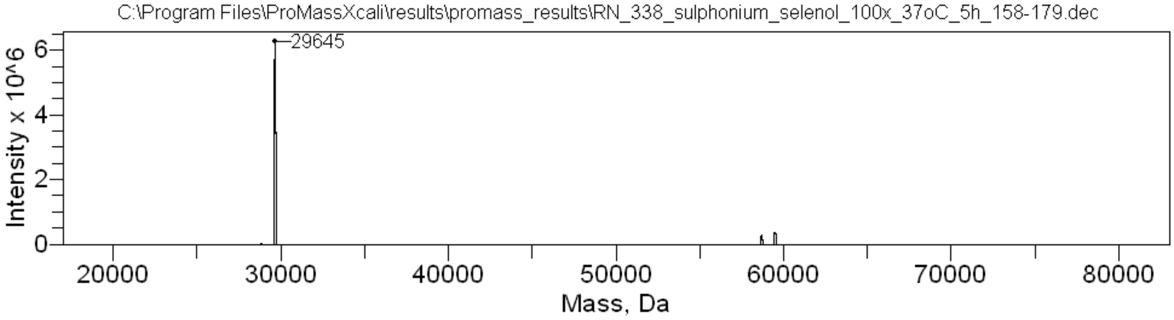

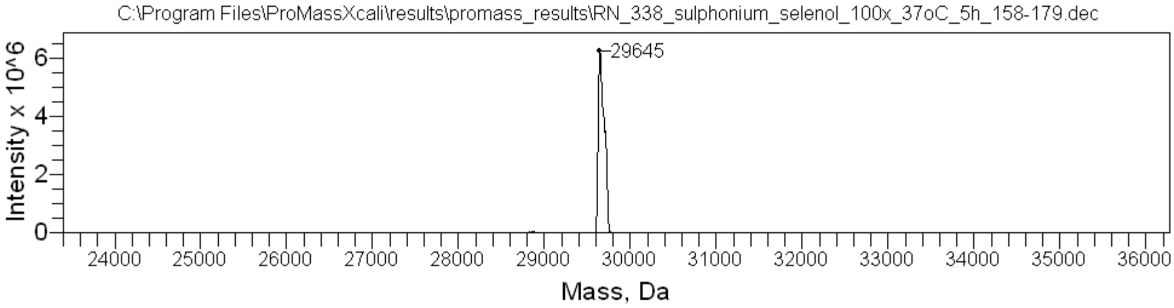

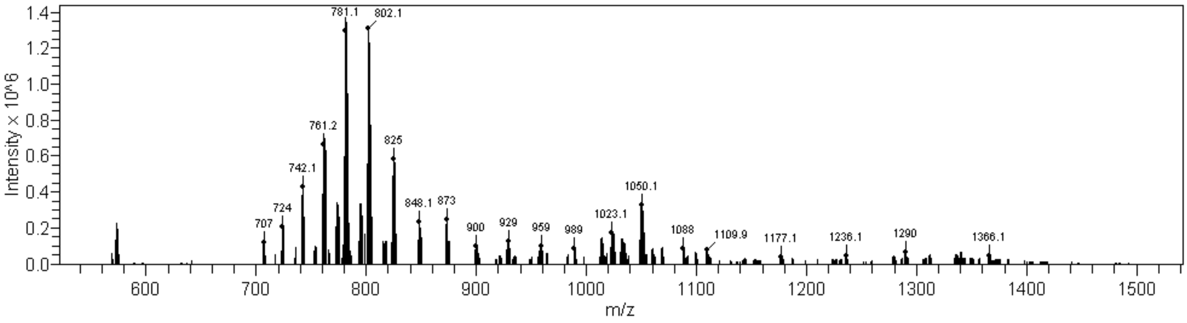

Reaction of sulphonium 8 with phthalimide

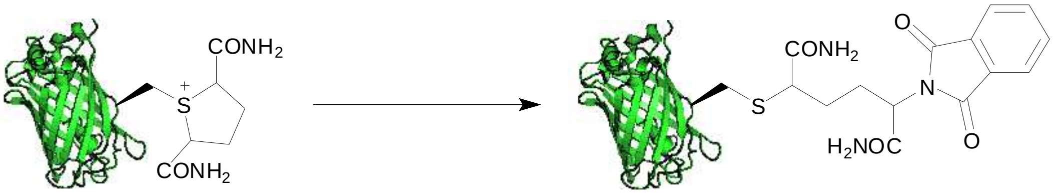

8 Calculated mass = 29486

10e Calculated mass = 29633

Potassium phthalimide (10 μL, 340 mM solution in DMF, 1000 equivalents) was added to a solution of sulfonium **8** (100 μL, 1.0 mg/mL) in sodium phosphate (100 mM, pH 8.0) at 21 °C. The mixture was vortexed for 1s, maintained at 37 °C for 2.5 h and analysed by LCMS.

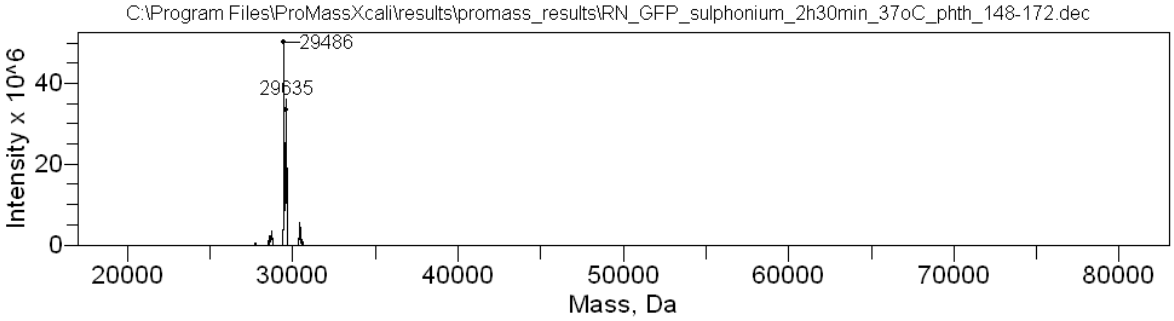

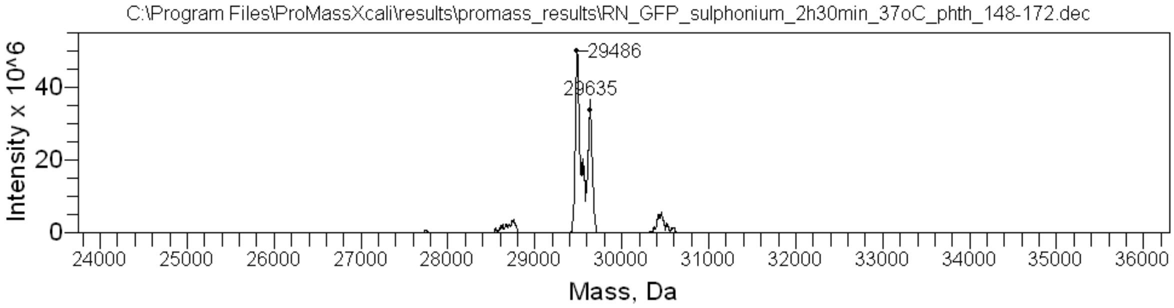

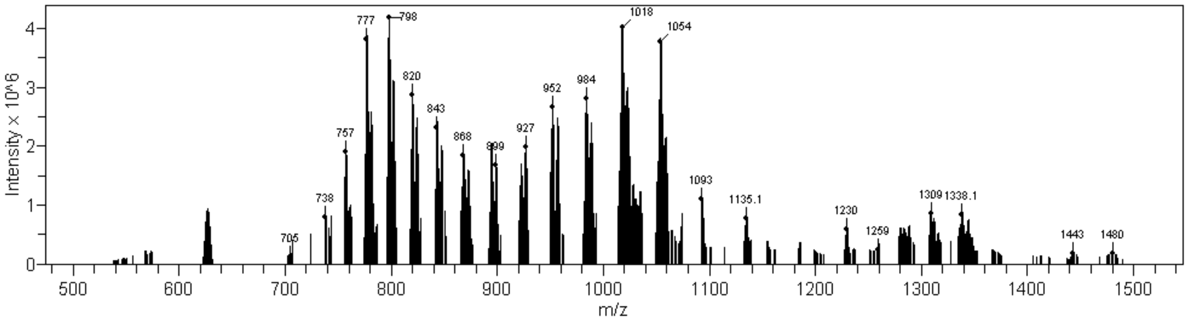

Reaction of sulphonium 8 with azide

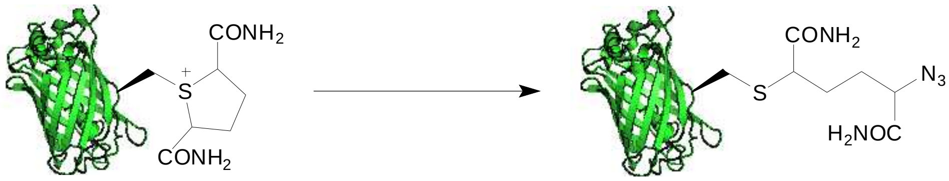

8 Calculated mass = 29486

10f Calculated mass = 29528

Sodium azide (10 µL, 340 mM solution in water, 1000 equivalents) was added to a solution of sulfonium **8** (100 µL, 1.0 mg/mL) in sodium phosphate (100 mM, pH 8.0) at 21 °C. The mixture was vortexed for 1s, maintained at 37 °C for 2.5 h and analysed by LCMS.

(Expected Mass : 29528, Observed Mass : 29528)

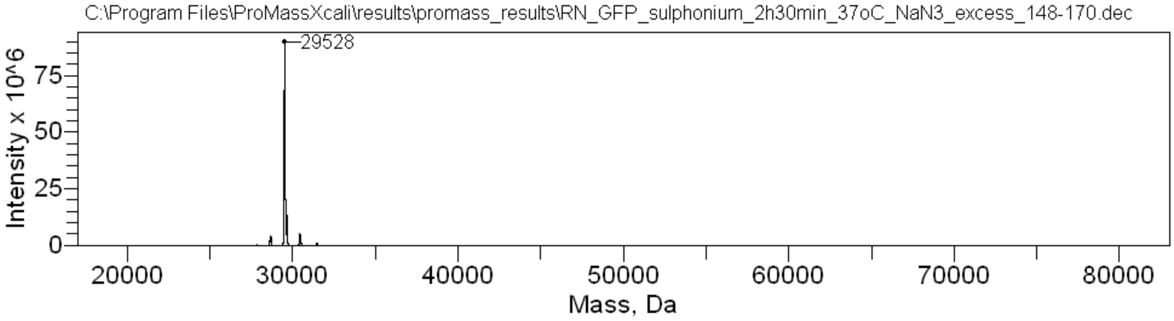

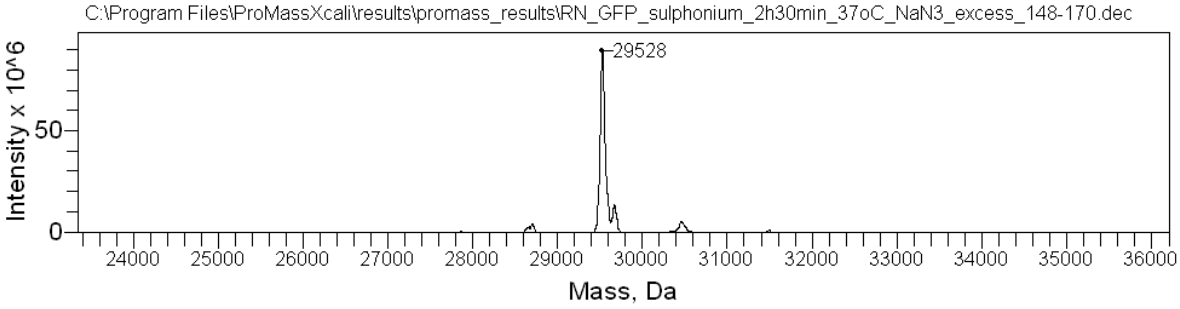

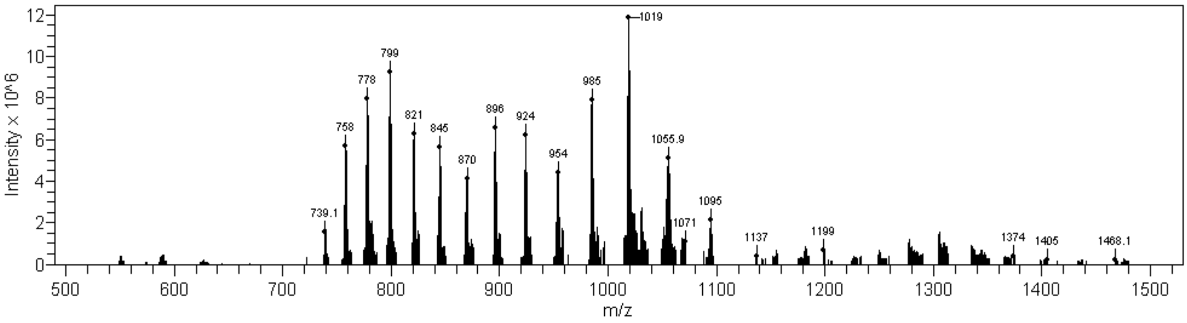

References

1. J. M. Chalker, S. B. Gunnoo, O. Boutureira, S. C. Gerstberger, M. Fernández-González, G. J. L. Bernardes, L. Griffin, H. Hailu, C. J. Schofield and B. G. Davis, *Chem. Sci.*, 2011, **2**, 1666.
2. P. Moody, M. E. B. Smith, C. P. Ryan, V. Chudasama, J. R. Baker, J. Molloy and S. Caddick, *ChemBioChem*, 2011, 1-3.
